# Liver Aging Index: A Noninvasive Score for Liver Biological Aging and Liver‐Related Outcomes in Multicohorts

**DOI:** 10.1111/acel.70565

**Published:** 2026-05-31

**Authors:** Zhiyu Wu, Shanshan Wu, Shuyao Song, Yating Huang, Canqing Yu, Dianjianyi Sun, Pei Pei, Ling Yang, Yiping Chen, Huaidong Du, Robin Walters, Iona Millwood, Hao Xu, Xiaoming Yang, Junshi Chen, Seung Up Kim, Salvatore Petta, Atsushi Nakajima, Emmanuel Tsochatzis, Jérôme Boursier, Elisabetta Bugianesi, Wah‐Kheong Chan, Manuel Romero‐Gomez, José Luis Calleja, Victor de Lédinghen, Laurent Castéra, Arun J. Sanyal, George Boon‐Bee Goh, Philip Noel Newsome, Jian‐Gao Fan, Michelle Lai, Xiao‐Dong Zhou, Zhengming Chen, Jun Lv, Liming Li, Vincent Wai‐Sun Wong, Ming‐Hua Zheng, Yuanjie Pang, the China Kadoorie Biobank Collaborative Group and VCTE‐Prognosis Study Group

**Affiliations:** ^1^ Department of Epidemiology & Biostatistics, School of Public Health Peking University Beijing China; ^2^ Department of Gastroenterology, Beijing Friendship Hospital Capital Medical University, State Key Laboratory of Digestive Health, National Clinical Research Center for Digestive Diseases Beijing China; ^3^ Center for Public Health and Epidemic Preparedness & Response Peking University Beijing China; ^4^ Ministry of Education Key Laboratory of Epidemiology of Major Diseases (Peking University) Beijing China; ^5^ Clinical Trial Service Unit & Epidemiological Studies Unit (CTSU), Nuffield Department of Population Health University of Oxford Oxford UK; ^6^ Tongxiang Center for Disease Control and Prevention Tongxiang Zhejiang China; ^7^ China National Center for Food Safety Risk Assessment Beijing China; ^8^ Department of Internal Medicine Yonsei University College of Medicine Seoul Republic of Korea; ^9^ Sezione di Gastroenterologia, Di.Bi.M.I.S. University of Palermo Palermo Italy; ^10^ Department of Gastroenterology and Hepatology Yokohama City University Graduate School of Medicine Yokohama Japan; ^11^ Royal Free Hospital University College London Institute for Liver and Digestive Health London UK; ^12^ Hepato‐Gastroenterology and Digestive Oncology Department Angers University Hospital Angers France; ^13^ Department of Medical Sciences, Division of Gastroenterology and Hepatology, A.O. Città Della Salute e Della Scienza di Torino University of Turin Turin Italy; ^14^ Gastroenterology and Hepatology Unit, Department of Medicine, Faculty of Medicine University of Malaya Kuala Lumpur Malaysia; ^15^ UCM Digestive Diseases, Virgen del Rocío University Hospital, Centro de Investigación Biomédica en Red en Enfermedades Hepáticas y Digestivas (CIBEREHD), Institute of Biomedicine of Seville (IBiS) University of Seville Seville Spain; ^16^ Department of Gastroenterology and Hepatology Hospital Universitario Puerta de Hierro Majadahonda Madrid Spain; ^17^ Echosens Paris France; ^18^ Université Paris Cité, UMR1149 (CRI), INSERM Paris France; ^19^ Hôpital Beaujon, Assistance Publique‐Hôpitaux de Paris (AP‐HP) Service D'hépatologie Clichy France; ^20^ Division of Gastroenterology, Hepatology and Nutrition, Department of Internal Medicine Virginia Commonwealth University School of Medicine Richmond Virginia USA; ^21^ Department of Gastroenterology and Hepatology Singapore General Hospital Singapore Singapore; ^22^ Institute of Hepatology, Faculty of Life Sciences & Medicine King's College London and King's College Hospital London UK; ^23^ Department of Gastroenterology and Hepatology, School of Medicine Shanghai Jiao Tong University Shanghai China; ^24^ Division of Gastroenterology & Hepatology Beth Israel Deaconess Medical Center, Harvard Medical School Boston Massachusetts USA; ^25^ MAFLD Research Center, Department of Hepatology The First Affiliated Hospital of Wenzhou Medical University Wenzhou China; ^26^ State Key Laboratory of Vascular Homeostasis and Remodeling Peking University Beijing China; ^27^ Medical Data Analytics Centre, Department of Medicine and Therapeutics The Chinese University of Hong Kong Hong Kong China; ^28^ State Key Laboratory of Digestive Disease, Institute of Digestive Disease The Chinese University of Hong Kong Hong Kong China; ^29^ Institute of Hepatology Wenzhou Medical University Wenzhou China; ^30^ Key Laboratory of Diagnosis and Treatment for the Development of Chronic Liver Disease in Zhejiang Province Wenzhou China

**Keywords:** biological age, liver aging, liver‐related outcome

## Abstract

Biological aging is a key determinant of liver disease and mortality, but there is little evidence on noninvasive index for assessment of liver biological aging. We developed the Liver Aging Index (LAI) in the China Kadoorie Biobank (CKB, *N* = 21,629) using Cox‐Gompertz proportional hazards model. The LAI incorporated three clinical factors (body mass index, systolic and diastolic blood pressure), eight plasma biomarkers (glucose, total cholesterol, triglycerides, high‐ and low‐density lipoprotein cholesterol, alanine aminotransferase, aspartate aminotransferase, and γ‐glutamyl transpeptidase), and two imaging biomarkers (fat attenuation parameter and liver stiffness measurement). External validation was conducted in the National Health and Nutrition Examination Survey (NHANES; *N* = 3412) and the VCTE‐Prognosis cohort (*N* = 12,170, 16 global centers). Across all cohorts, the LAI demonstrated strong discrimination for all‐cause mortality (AUROC: 0.764 in NHANES; 0.759 in VCTE‐Prognosis), outperforming chronological age (*p* < 0.05). Liver aging acceleration (LAA), defined as the difference between LAI and chronological age, was associated with substantially elevated risks: each 1‐SD increase in LAA conferred a 22%–85% higher risk of all‐cause mortality and a 34%–170% higher risk of liver‐related event or mortality. Using genetic instruments identified in CKB, we found genetic predisposition to accelerated liver aging was associated with higher risks of cirrhosis and liver cancer (HR = 3.94 [3.20–4.86] and 7.82 [2.05–29.80]), further validated in Biobank Japan. Integrating genetics and proteomics revealed novel pathophysiological involvement of amyloid‐beta clearance pathway and amyloid precursor protein in liver aging. These findings demonstrate the feasibility of a noninvasive, liver‐specific biological aging index and provide new insights into mechanisms underlying liver aging.

## Graphical abstract

A noninvasive biomarker–based Liver Aging Index accurately predicts liver‐related events and mortality across diverse populations, offering a practical and generalizable tool with strong potential for clinical and public health application.

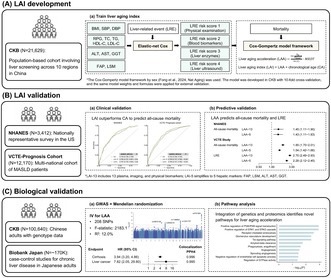

**Abbreviations**
APP: amyloid precursor proteins
AUROC: area under the receiver operating characteristic curves
BA: biological age
CA: chronological age
CKB: China Kadoorie Biobank
GWAS: genome‐wide association study
ICD‐10: 10th International Classification of Diseases
LAA: liver aging acceleration
LAI: Liver Aging Index
LRE: liver‐related event
LRM: liver‐related mortality
MAE: mean absolute error
MASLD: metabolic dysfunction‐associated steatotic liver disease
NHANES: National Health and Nutrition Examination Survey
RMSE: root mean square error
VCTE: Vibration‐Controlled Transient Elastography

## 1 Introduction

Chronic liver disease is an age‐related disease with the incidence rate increasing quadratically with age, and it has posed a substantial disease burden globally. According to the Global Burden of Disease Study in 2023, there were approximately 1.6 billion prevalent cases of chronic liver disease globally, of which 77% were attributable to nonalcoholic fatty liver disease (IHME 2025). China accounted for 25% of the global prevalent cases of chronic liver disease (IHME 2025), and a recent nationwide study estimated the prevalence of cirrhosis in Chinese adults at 0.87%, increasing to 1.64% in those aged 60 years or older (Man et al. 2023). Aging promotes pathological changes in the liver, which are key risk factors for liver steatosis, fibrosis, and ultimately, liver cancer. Meanwhile, as a central organ regulating metabolic and immune processes, the liver plays a pivotal role in maintaining systemic homeostasis. Age‐related deterioration of hepatic structure and function may disrupt these processes through multiple pathways including metabolic reprogramming, oxidative stress, and inflammation, thereby contributing to systemic aging (Zhong et al. 2025). The liver biological age, constructed from quantifiable age‐related liver biomarkers, may serve as an assessment tool for both liver‐specific and systemic aging, and may inform strategies on early detection of aging and the prevention and control of chronic liver disease.

Several studies, mostly in Western populations, have constructed biological aging indexes specific to the liver. These studies have shown moderate to high correlations between liver biological age (BA) and chronological age (CA). However, these studies have trained liver BA against CA, without incorporating age‐related diseases such as all‐cause mortality and liver‐related outcomes. Therefore, the clinical utility of these liver biological indexes in predicting future age‐related diseases has been limited. Previous studies are also limited due to a lack of external validation for the liver aging model (Moqri et al. 2024), especially from multiethnic and multinational settings, and the transportability of liver biological age has not been investigated. Lastly, recent studies have developed proteomics aging clocks and selected proteins with differential expression in the liver to construct “Liver Protein Age” (Goeminne et al. 2025), but the high price for proteomics assays hinders the feasibility of applying these organ‐specific proteomics clocks to larger populations.

Therefore, the objectives of the current study were: (1) to develop a novel, noninvasive index (i.e., the Liver Aging Index, LAI) for assessment of liver biological aging involving blood‐based biomarkers, imaging biomarkers, and clinical phenotypes in the general population (the China Kadoorie Biobank [CKB]); (2) to validate the risk stratification performance of the LAI for systemic aging (i.e., all‐cause mortality) and liver‐related outcomes (liver‐related event [LRE] and liver‐related mortality [LRM]) in the general population (National Health and Nutrition Examination Survey [NHANES]) and adults with metabolic dysfunction‐associated steatotic liver disease (MASLD) (Vibration‐Controlled Transient Elastography [VCTE]‐Prognosis cohort); (3) to evaluate the associations of LAI with two geriatric indicators, including frailty and multimorbidity; and (4) to examine the potential causal associations of the LAI with all‐cause mortality and liver‐related outcomes utilizing genetic instruments for the LAI in East Asians (Chen et al. 2025).

## 2 Methods

### 2.1 Study Design and Participants

The study adhered to the Transparent Reporting of a multivariable prediction model for Individual Prediction or Diagnosis (TRIPOD) guidelines (Table S1) (Collins et al. 2015). All participants provided informed written consent before inclusion, and research ethical approvals were obtained for all study cohorts. For CKB, NHANES, and the VCTE‐Prognosis cohort, details on data collection, physical examinations, blood biochemistry assays, and ascertainment for incidence and mortality were reported in the Supporting Information.

### 2.2 Development Cohort

First, we developed the LAI using data from the 3rd resurvey of CKB (Chen et al. 2011). Briefly, the CKB baseline survey recruited 512,724 participants from 10 (5 urban and 5 rural) diverse areas in China during 2004–2008. After the baseline survey, approximately 5% of surviving cohort members from each of the 10 study regions were randomly selected to participate in resurveys every 5–6 years. The 3rd resurvey was conducted between August 2020 and December 2021 and included additional assessments of liver steatosis and fibrosis by transient elastography (FibroTouch [Hisky, Wuxi, China]) (Man et al. 2023). For model derivation, we included the 25,087 participants in the 3rd follow‐up resurvey and excluded: (1) missing or invalid measurement of LAI features including liver enzymes and transient elastography; (2) those with a diagnosis of cancer, cirrhosis or chronic hepatitis or missing baseline medical history. Finally, a total of 21,629 participants were retained for the main analysis, with ages ranging from 45 to 95 years.

### 2.3 Validation Cohorts

Next, model performance of the LAI was externally validated in two independent datasets: the US NHANES and a multicenter VCTE‐Prognosis cohort. The NHANES is a nationally representative survey of the noninstitutionalized US population. For external validation in the general population of middle‐aged and older adults, we included the 2017–2018 cycle of 9254 individuals because VCTE exams (FibroScan, model 502 V2 Touch [Echosens, Waltham, MA]) were added to this cycle, with the following exclusion criteria: (1) participants < 45 years of age or pregnant to align the age range of the study population of the development CKB cohort; (2) those with self‐reported diagnosis of liver cancer, cirrhosis, or chronic hepatitis before baseline. After these exclusions, 3412 individuals aged ≥ 45 years were retained for the main analysis.

The VCTE‐Prognosis cohort is a multinational, prospective cohort involving 18,057 participants with clinical diagnosis of MASLD who underwent VCTE examinations at tertiary referral centers in the US, Europe, and Asia from February 2004 to January 2023. Detailed information on the VCTE‐Prognosis cohort has been presented in previously published studies (FibroScan [Echosens, Paris, France]) (Lin et al. 2024; Zhou et al. 2024). The VCTE‐Prognosis cohort was used as an external validation in middle‐aged and older adults with MASLD, with the following exclusion criteria: (1) those aged < 45 years to align the age range of the study population of the development CKB cohort; (2) those with invalid VCTE results; (3) those with invalid blood biochemistry; and (4) those with self‐reported diagnosis of cancer, cirrhosis, or chronic hepatitis. After these exclusions, 12,170 individuals were retained for the main analysis.

### 2.4 Definitions of All‐Cause Mortality and Liver‐Related Outcomes

In CKB, the vital status of each participant was determined periodically through the China CDC's Disease Surveillance Points (DSP) system and the national health insurance system, supplemented by regular checks against local residential and health insurance records and by annual active confirmation through street committees or village administrators (Yang et al. 2006). Follow‐up was conducted through December 31, 2023. For NHANES 2017–2018, public‐use Linked Mortality Files (LMF) provide all‐cause mortality follow‐up from survey participation through December 31, 2019, for adult participants. In the VCTE‐Prognosis cohort, the outcomes of interest included incident all‐cause mortality through Jul 31, 2023.

For evaluation of liver‐related outcomes, incident LRE and LRM during follow‐up period were the main outcomes of interest. LREs were defined by major clinical complications of cirrhosis and advanced chronic liver diseases: (1) hepatocarcinogenesis: hepatocellular carcinoma; (2) portal hypertension‐related event: variceal bleeding and ascites; (3) liver dysfunction‐related events: hepatic failure/acute‐on‐chronic liver failure, overt hepatic encephalopathy, and other severe conditions. A detailed list of 10th International Classification of Diseases (ICD‐10) codes is shown in Table S2. LRM was defined with the same ICD‐10 codes as the underlying cause of death. In CKB, the morbidity and mortality of participants were ascertained via linkages to the death registry, disease registry, and health insurance records, with censoring date until Dec 31, 2023. The CKB study has ensured the accuracy of diagnoses through outcome adjudication, with diagnostic accuracy ranging from 88% to 96% for major diseases (e.g., coronary heart disease, stroke, diabetes, cancer, and chronic respiratory pulmonary disease) (Im et al. 2023) and ~90% for other diseases (e.g., liver disease and inflammatory bowel disease) (Song et al. 2024; Pang et al. 2018). In NHANES, participants were matched to mortality status through December 31, 2019 using the NHANES Public Use Linked Mortality File. In VCTE‐Prognosis cohort, the mortality and LRE diagnosis of the events was based on prospective follow‐up, medical record review, or validated registries until Jul 31, 2023 with positive predictive values of at least 90% (Lin et al. 2024).

### 2.5 Statistical Analysis

#### 2.5.1 Model Development

We developed the LAI using a two‐step modeling framework. Briefly, we constructed the LAI within a biological age modeling framework based on the Biomarkers of Aging Consortium Roadmap approach (Goeminne et al. 2025), grouped biomarkers according to recommendations by the Aging Biomarker Consortium on liver aging biomarkers (Levine et al. 2018), and applied a Gompertz‐based modeling strategy following the second‐generation biological aging models (Fong et al. 2024; Xing et al. 2023).

In step 1, we grouped the 13 biomarkers into 4 categories by constructing four dimensional LRE risk scores based on elastic‐net Cox regression, with LRE as the outcome. The model training was conducted using 10‐fold cross‐validation to identify the optimal regularization parameter. For each participant, linear predictors of the LRE risk score were derived from the optimized models. These risk scores captured the composite effect of multiple biomarkers on LRE risk within each category and also accounted for the colinearity among biomarkers.

In step 2, we employed a Cox‐Gompertz framework to construct the LAI based on second‐generation biological age clocks with mortality as the outcome (Levine et al. 2018; Fong et al. 2024). The null model included CA alone to estimate baseline mortality hazard ($h_{0}$) and sex‐specific mortality rate doubling time (MRDT). The liver biological age model with 13 biomarkers incorporated LRE risk scores to estimate mortality hazard.

(1) $$\text{Liver aging acceleration}\;\left(\mathrm{LAA}-13\right)=\frac{\ln\left(\frac{h_{\text{liver}}}{h_{0}}\right)}{\ln\left(2\right)}\cdot \text{MRDT}$$

For each participant, Liver Aging Acceleration (LAA) was quantified as the equivalent years of accelerated liver aging associated with increased mortality risk: a higher LAA reflects accelerated liver aging, whereas lower values indicate delayed liver aging. The LAI was calculated by integrating LAA with CA. Participants were further classified into ‘liver aging acceleration’ and ‘liver aging deceleration’ groups based on LAA quartiles, with the highest quartile representing accelerated liver aging (LAI high) and the lowest quartile representing decelerated liver aging (LAI low).

To enhance clinical transportability, we developed a simplified version of LAI (i.e., LAI‐5) focusing exclusively on five hepatic biomarkers directly linked to liver function and fibrosis, including fat attenuation parameter, liver stiffness measurement, alanine aminotransferase, aspartate aminotransferase, and gamma‐glutamyl transpeptidase, using the same Cox‐Gompertz framework stratified by sex. Details of model development and validation were reported in the Supporting Information.

#### 2.5.2 Model Assessment and Validation

Model assessment for internal validation was performed using 10‐fold cross‐validation within the CKB training set, along with independent external validation in the NHANES and VCTE‐Prognosis cohorts. For assessment, the estimated LAI values were compared with CA using Pearson's correlation coefficients, root mean square errors (RMSEs), and mean absolute error (MAE) to evaluate accuracy in both the derivation and independent validation cohorts. For validation, there were 3 steps. First, we examined whether LAI showed better predictive performance for all‐cause mortality and liver‐related outcomes compared with CA. The area under the receiver operating characteristic curves (AUROC) was calculated and the comparison between LAI and CA was done by Delong's test. Second, we examined whether LAA could predict future risks of all‐cause mortality and liver‐related outcomes using Cox proportional hazards models, adjusting for age, sex, regions (in CKB and VCTE‐Prognosis cohort), race/ethnicity (in NHANES), education, and marital status. LRM was not examined in the VCTE‐Prognosis cohort because of the limited number of cases. LRM and LRE were not examined in NHANES because the underlying causes of death were unavailable. Moreover, stratified analysis by MASLD status (with or without) was conducted to further substantiate the predictive performance of LAA. Third, we examined the associations of LAI and LAA with two geriatric indicators, frailty and multimorbidity, in the CKB cohort. Associations were estimated using binary or multinomial logistic regression, as appropriate. Fourth, to investigate the potential causal associations and biological mechanisms, we performed a genome‐wide association study (GWAS) of LAA in CKB participants and conducted Mendelian randomization to examine genetic associations of LAA with all‐cause mortality and liver‐related outcomes. The genetic associations in CKB were further validated by colocalization in the Biobank Japan. Finally, Mendelian randomization was performed in CKB to examine the causal impact of accelerated liver aging on circulating protein biomarkers and to identify potential novel pathways underlying liver aging.

All statistical analyses were performed using R software version 4.3.2. A *p*‐value < 0.05 was considered statistically significant unless otherwise specified.

## 3 Results

### 3.1 Characteristics of Study Participants

The characteristics of the study participants from three cohorts were detailed in Table 1. The mean age ranged from 59.1 years to 65.3 years, and the proportion of female participants ranged from 48.1% to 64.6%. Diabetes was more prevalent in the VCTE‐Prognosis validation cohort compared to CKB derivation cohort and NHANES validation dataset. Participants in the VCTE‐Prognosis validation cohort had higher LSM and liver enzyme levels, while those in the NHANES validation cohort had a higher BMI.

**TABLE 1:** Baseline characteristics of study participants.

	Derivation cohort	Validation cohorts	
	CKB (*N* = 21,629)	NHANES (*N* = 3412)	VCTE‐Prognosis cohort (*N* = 12,170)
Age, years	65.3 (9.0)	63.2 (10.6)	59.1 (8.6)
Female	13,963 (64.6)	1733 (50.8)	5850 (48.1)
BMI, kg/m^2^	24.5 (3.5)	29.9 (7.0)	27.9 (6.7)
SBP, mmHg	133.3 (21.0)	133.4 (20.7)	130.3 (18.7)
DBP, mmHg	78.4 (10.6)	72.8 (13.5)	78.6 (12.2)
Diabetes	3418 (15.8)	437 (12.8)	4947 (40.6)
RPG, mmol/L	6.5 (2.6)	6.1 (2.4)	6.6 (2.2)
AST, U/L	24.9 (12.0)	22.2 (12.7)	38.3 (32.1)
ALT, U/L	23.7 (15.9)	21.5 (14.4)	44.3 (44.3)
GGT, U/L	30.7 (36.7)	34.6 (47.5)	73.6 (127.3)
TC, mmol/L	5.1 (1.4)	4.9 (1.1)	4.7 (1.1)
TG, mmol/L	2.0 (1.5)	1.7 (1.3)	1.8 (1.2)
LDL‐C, mmol/L	2.8 (1.4)	2.8 (1.0)	2.7 (1.0)
HDL‐C, mmol/L	1.4 (0.4)	1.4 (0.4)	1.3 (0.4)
FAP, dB/m^a^	248.9 (34.7)	275.3 (60.7)	300.9 (42.6)
LSM, kPa	7.4 (2.6)	6.4 (5.8)	8.3 (7.3)

*Note:* Values are presented as mean (standard deviation) for continuous variables and number (percentage) for categorical variables. The NHANES and VCTE‐P validation cohorts included participants aged 45–95 years, consistent with the age range of the CKB derivation cohort.

Abbreviations: FAP, fibrosis attenuation parameter; LSM, liver stiffness measurement.

^a^ For FAP measurements, the ultrasound attenuation parameter was used in CKB, and the controlled attenuation parameter was used in NHANES and the VCTE‐Prognosis cohort.

### 3.2 Characteristics of LAI

**FIGURE 1:**
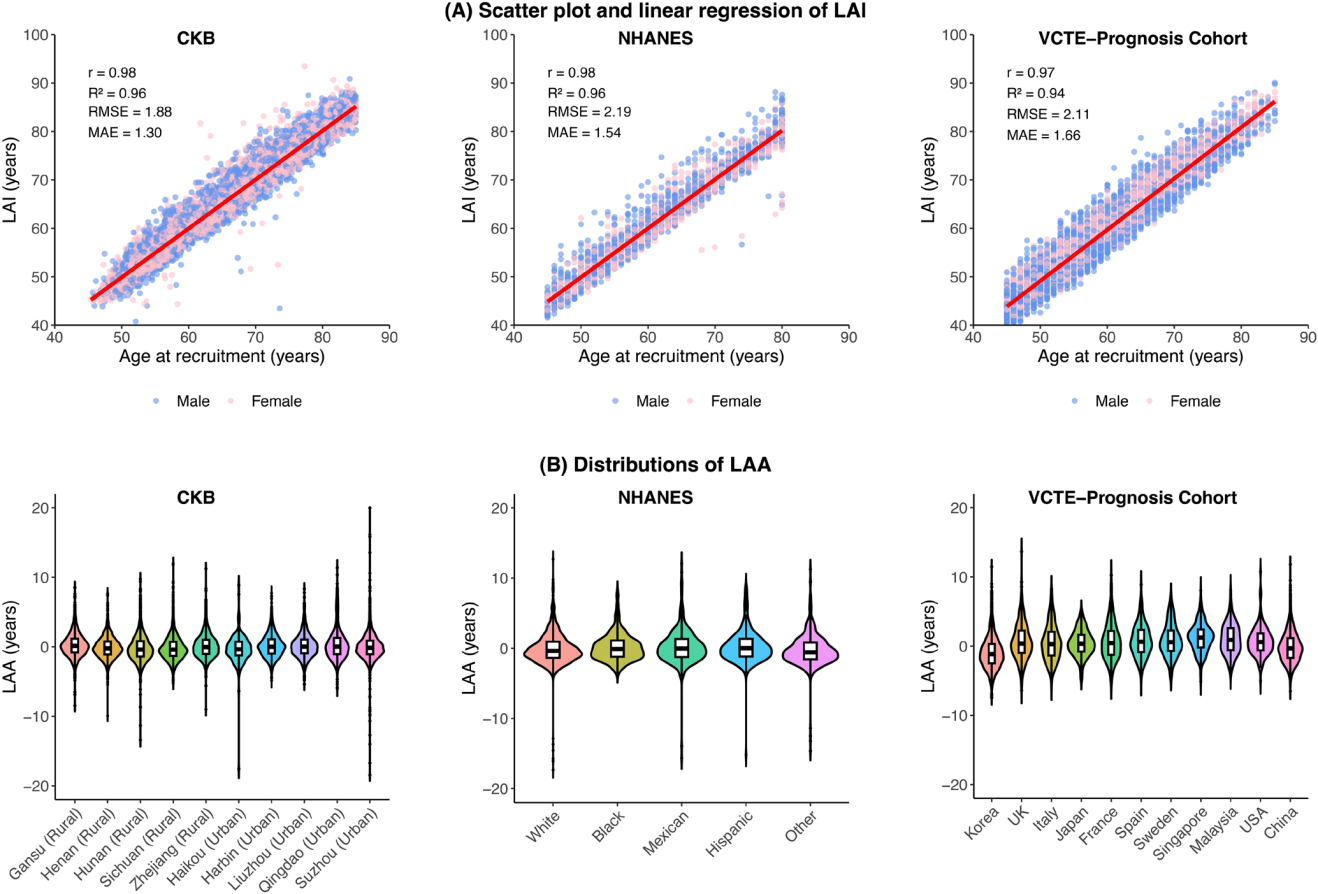
Metrics of LAI in the development and validation cohorts. Panel A shows scatter plot and linear regression of CA versus LAI for men (blue) and women (red). In CKB and external validation cohorts, LAI is strongly correlated with CA (*r* = 0.97–0.98, *R* ^2^ = 0.94–0.96). Panel B shows distributions of LAA according to 10 geographic regions of residence in CKB, self‐reported race in NHANES, and the country of the participating center in VCTE‐Prognosis cohort. Violin plots with center line, box limits and whiskers representing the median, interquartile range, and minima/maxima within each group, respectively. MAE, mean absolute error; RMSE, root mean squared error.

The correlation structure among 13 liver‐related biomarkers was shown in Figure S1. Both LAI‐13 and LAI‐5 showed strong alignment with CA across the derivation and validation cohorts (Figure 1, Table S3, and Figure S2). In the CKB training cohort, the mean (±SD) of CA and LAI‐13 was 65.27 ± 9.02 years and 65.28 ± 9.23 years, respectively. LAI‐13 was strongly correlated with CA (*r* = 0.98), with a low RMSE of 1.88 and MAE of 1.30. Similar high correlations were observed in the NHANES and VCTE‐Prognosis cohort, with correlation coefficients of 0.98 and 0.97, RMSE of 2.19 and 2.11, as well as MAE of 1.54 and 1.66, respectively. For ease of external validation and practical application, an online calculator for the LAI has been made publicly available at https://liveragingindex.github.io.

### 3.3 Validation of LAI

**FIGURE 2:**
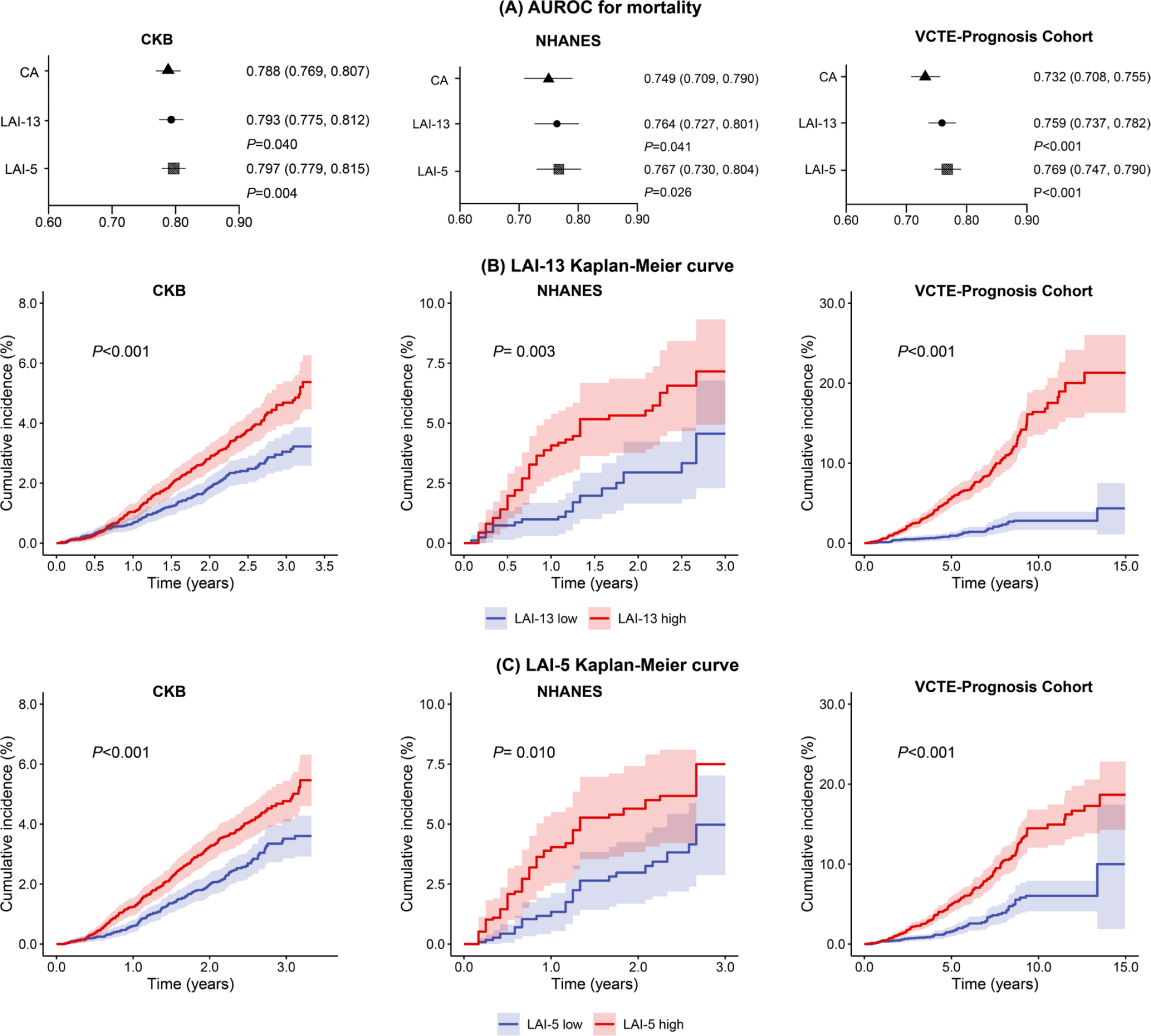
AUROC and Kaplan–Meier curves for all‐cause mortality in the development and validation cohorts. Panel A shows AUROCs of the LAI versus CA using DeLong's test. Panel B and C show Kaplan–Meier survival curves where subjects were classified into a LAI high group (LAA > Q3) and a LAI low group (LAA < Q1) based on quartile distribution of LAA. The analyses were adjusted for age, sex, regions (CKB and VCTE‐Prognosis), race/ethnicity (NHANES), education, and marital status. All *p* values were calculated using the log‐rank test for comparing survival distributions between groups. CA, chronological age; LAI, liver aging index.

In the CKB cohort, internal validation with 10‐fold cross‐validation showed that LAI‐13 and LAI‐5 achieved an AUROC of 0.793 and 0.797 for all‐cause mortality, respectively, significantly outperforming CA (AUROC = 0.788, *p* < 0.05, Figure 2). Similar results were observed in the NHANES external validation cohort and the VCTE‐Prognosis cohort with MASLD patients, with LAI consistently yielding higher AUROC than CA (both *p* < 0.05, Figure 2). Regarding LRE and LRM, LAI demonstrated superior discriminative ability compared with CA. This improvement in discrimination was accompanied by only modest gains in overall prediction error in the CKB cohort and consistent patterns were observed in the VCTE‐Prognosis cohort (Table S4, Figure S3). Kaplan–Meier curves demonstrated a clear separation between participants in the highest quartile (advanced LAI relative to CA) and those in the lowest quartile (younger LAI relative to CA), indicating that individuals with accelerated liver aging had substantially higher risks of mortality, LRE, and LRM (log‐rank *p* < 0.01 across all cohorts, Figures 2 and S3).

### 3.4 Associations of LAA With All‐Cause Mortality, Liver‐Related Outcomes, and Geriatric Indicators

**FIGURE 3:**
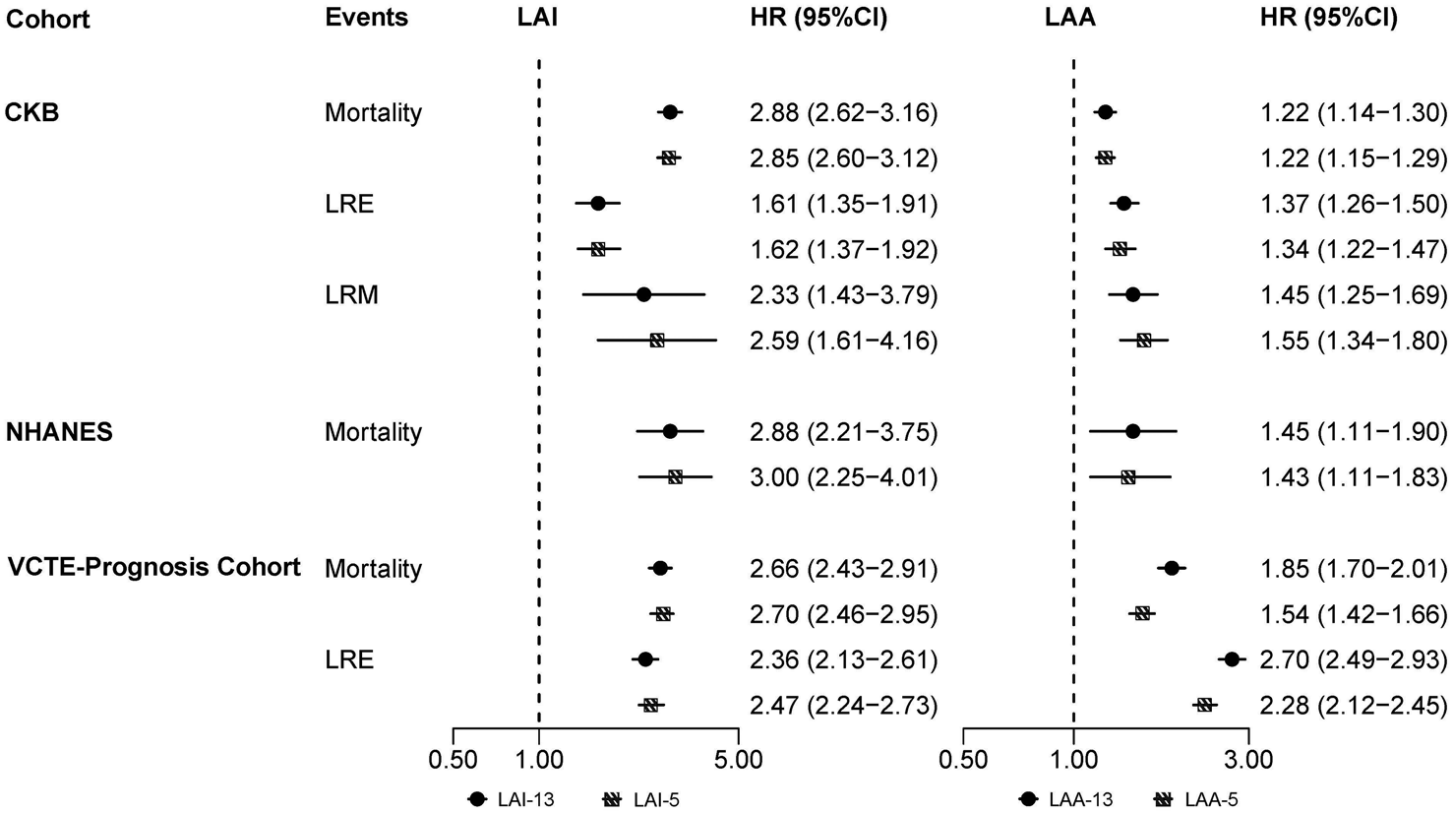
Associations of LAI with all‐cause mortality and liver‐related outcomes in the development and validation cohorts The results are obtained through Cox regression model calculation in CKB and VCTE‐Prognosis cohorts, and weighted Cox regression model calculation in NHANES, adjusting for age, sex, regions (CKB and VCTE‐Prognosis), race (NHANES), education, and marital status. LAA, liver aging acceleration; LAI, liver aging index.

The LAA was consistently associated with all‐cause mortality, LRE, and LRM (Figure 3). For all‐cause mortality, in CKB, each 1‐SD increase in LAA‐13 and LAA‐5 was both associated with a 22% higher risk of all‐cause mortality (HR for LAI‐13: 1.22 [1.14, 1.30]; HR for LAI‐5: 1.22 [1.15, 1.29]). Consistent associations were observed in the NHANES validation cohort, where each 1‐SD increase in LAA‐13 was associated with a 45% higher risk of all‐cause mortality (HR [95% CI]: 1.45 [1.11, 1.90]) and LAA‐5 yielded comparable results (HR [95% CI]: 1.43 [1.11, 1.83]). In the VCTE‐Prognosis cohort, both LAA‐13 and LAA‐5 were associated with higher mortality risk, with HRs of 1.85 (1.70, 2.01) and 1.54 (1.42, 1.66) per 1‐SD increment, respectively. For LRE, higher LAA‐13 and LAA‐5 were associated with increased risk in both the CKB deviation cohorts and the VCTE‐Prognosis cohort (HRs ranging from 1.34 to 2.70 per 1‐SD increase; *p* < 0.05). Moreover, in CKB, higher LAA‐13 and LAA‐5 were associated with significantly increased risk of LRM, with HRs of 1.45 and 1.55 per 1‐SD increase, respectively (Figure 3).

Subgroup analyses in CKB showed broadly consistent associations of the LAI and LAA with all‐cause mortality and liver‐related outcomes across MASLD and non‐MASLD populations, with no significant heterogeneity between subgroups (*P* for heterogeneity > 0.05) (Figure S4).

Further analysis in CKB showed that both LAI and LAA were associated with frailty and multimorbidity (Figure S5). Both LAA‐13 and LAA‐5 were associated with frailty, with odds ratios (OR) of 1.18 (1.15, 1.22) and 1.02 (1.01, 1.05) for per 1‐SD increase, respectively. The associations of LAI and LAA with multimorbidity strengthened with increasing condition counts. For each SD increment of LAA‐13, ORs for having one, two, and three or more conditions were 1.01 (0.99, 1.03), 1.06 (1.03, 1.08), and 1.10 (1.08, 1.12), respectively. In contrast, LAA‐5 showed weaker associations with multimorbidity and was associated with having three or more conditions (OR [95% CI]: 1.02 [1.01, 1.03]).

### 3.5 Biological Mechanisms of LAI

**FIGURE 4:**
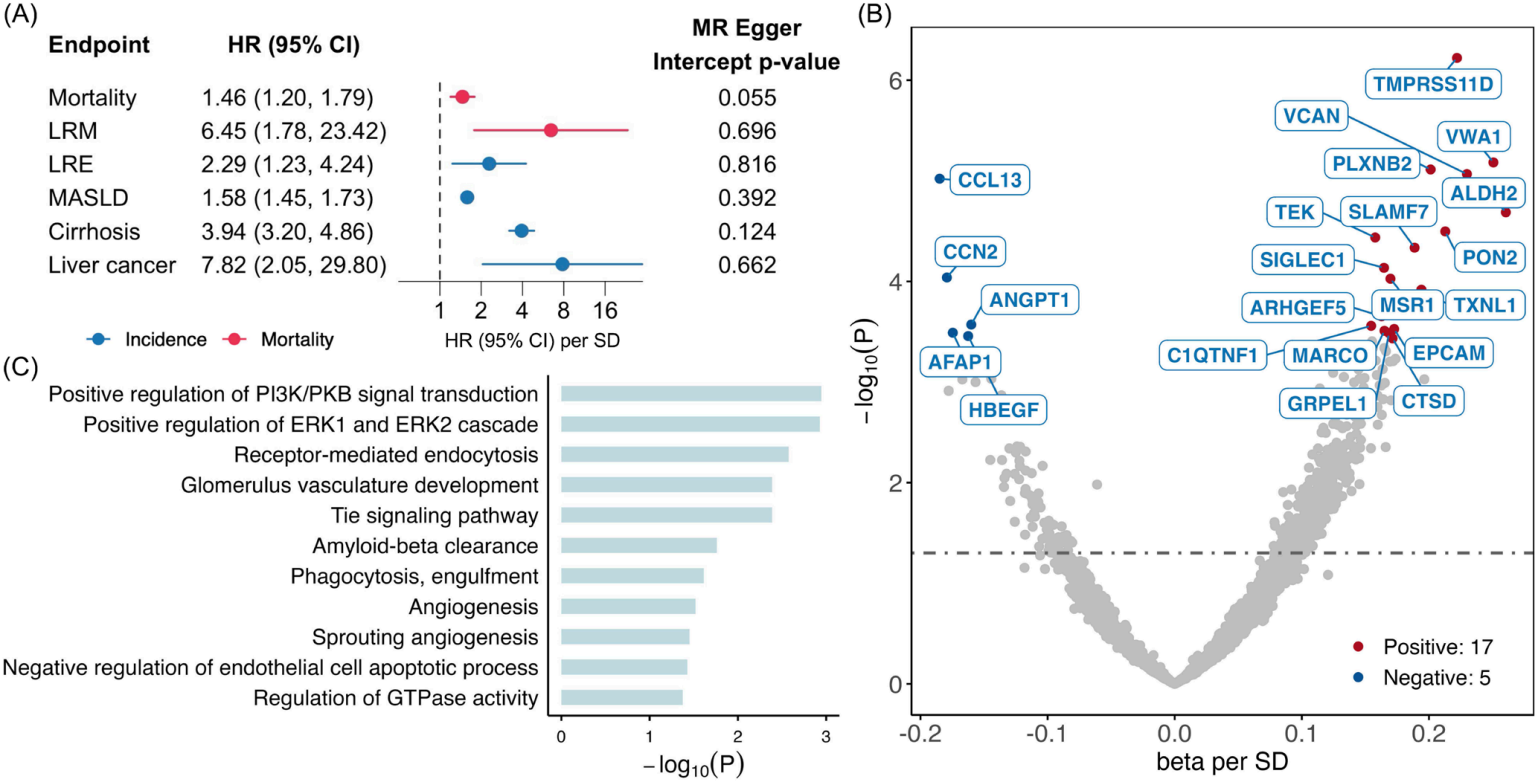
Biological validation of LAI in CKB. Panel A shows the genetic associations of LAA with all‐cause mortality and liver‐related outcomes in CKB. Panel B shows the genetic associations of LAA with 2923 circulation protein biomarkers where 22 proteins reach the statistical significance of false discovery rate < 0.05, with their names shown in boxes. Panel C shows results of pathway enrichment analyses including all 22 proteins in panel B.

Overall, 208 SNPs were identified for LAA and were used in Mendelian randomization. The instruments for LAA showed good performance, with an *F*‐statistic of 2183.1 and an *R* ^2^ of 12.0% and further details on the assumption assessment were shown in Supporting Information. Mendelian randomization analysis showed a positive association between genetically predicted LAA and all‐cause mortality (HR [95% CI]: 1.46 [1.20, 1.79]), with stronger associations observed for LRM and LRE (HRs of 6.45 [1.78, 23.42] and 2.29 [1.23, 4.24], respectively). Specifically, for MASLD, cirrhosis, and liver cancer, the genetically predicted LAA also demonstrated strong associations with all three outcomes, with HRs ranging from 1.58 to 7.82 (Figure 4A). The genetic associations of LAA with cirrhosis and liver cancer were further validated by colocalization in the Biobank Japan (posterior probability of H4 > 0.9, Table S5). We further explored the associations between genetically predicted LAA and a range of extrahepatic diseases. Genetically predicted LAA was found to be associated with cardiovascular diseases and their risk factors, including intracerebral hemorrhage, cerebral infarction, atrial fibrillation, and primary hypertension, as well as with gout (Table S6). In addition, genetically predicted LAA showed positive correlations with serum uric acid levels and inverse associations with amyloid precursor proteins (Table S6).

To explore potential biological mechanisms, Mendelian randomization was conducted to explore potential causal associations between LAA and the proteome involving 2923 circulating protein biomarkers. After multiple correction, we identified that genetically determined LAA was associated with 22 plasma proteins (false discovery rate < 0.05, Figure 4B, Table S7). Among these, 17 proteins were positively associated with genetically predicted LAA, including TMPRSS11D, PLXNB2, VCAN, VWA1, and ALDH2, while five proteins showed negative associations with LAA, including CCL13, CCN2, AFAP1, ANGPT1, and HBEGF. These proteins were enriched in signaling pathways such as positive regulation of phosphatidylinositol 3‐kinase/protein kinase B signaling, positive regulation of the ERK1 and ERK2 cascade, and receptor‐mediated endocytosis (Figure 4C).

## 4 Discussion

In the current study, we developed and validated the LAI, a novel, noninvasive index to reflect liver‐specific biological aging that integrated clinical phenotypes, blood‐based biomarkers and imaging data using Cox‐Gompertz model framework. We showed that LAI outperformed CA to predict all‐cause mortality and that LAA was associated with higher risks of all‐cause mortality as well as LRE and LRM. Utilizing the largest genetic studies in East Asia involving ~270 thousand participants (CKB and Biobank Japan), liver aging acceleration showed potential causal associations with liver‐related outcomes. Integrating genomics and proteomics data in CKB, we identified novel pathways underlying liver aging, including glomerulus vasculature development, sprouting angiogenesis, and amyloid‐beta clearance.

To the best of our knowledge, eight studies, primarily conducted in Western countries, have developed biological aging indexes specific for the liver (Table S8). Among these, three constructed indices using liver‐specific biomarkers, such as liver imaging parameters and liver enzymes, while the remaining five derived liver‐specific aging metrics within multiorgan biological aging frameworks by integrating multiomics data (e.g., proteomics, metabolomics, and DNA methylation). Consistent with our findings, most prior studies reported moderate to high correlations between liver biological age and chronological age, with correlation coefficients ranging from 0.40 to 0.85. However, the majority of these studies relied on first‐generation framework, lacked data on incidence and mortality of liver diseases, and did not conduct external validation.

For the three studies constructing liver biological age using liver‐specific biomarkers, the first‐generation model has been used with chronological aging as the training outcome (Xing et al. 2023; Tian et al. 2023; Le Goallec et al. 2022). Le Goallec et al. constructed the liver image age using liver magnetic resonance imaging data in 43,267 participants (37–82 years) as part of a Wide Association Study, however, they observed only weak correlations between liver aging acceleration and liver diseases (Pearson correlations: −0.015 to 0.008) (Le Goallec et al. 2022). Tian et al. constructed the hepatic age score using 9 blood biochemistry markers in 143,423 UK Biobank adults (39–73 years, mean age 56.7) and reported associations between liver aging acceleration and increased risks of all‐cause mortality, cancer and cardiovascular mortality as well as incident cirrhosis, but no external validation was conducted (Tian et al. 2023). The remaining five studies developed liver‐specific biological aging indices within the framework of multiorgan and multiomics (Goeminne et al. 2025; Ahadi et al. 2020; Sehgal et al. 2025; Nie et al. 2022; Oh et al. 2023). Of these, three used the first‐generation model (i.e., chronological aging as the training outcome) and two used the second‐generation model (i.e., all‐cause mortality as the training outcome). These studies evaluated the associations of liver age with various chronic diseases across multiple organ systems and reported relatively robust results. This supports the notion that liver age may also reflect systemic aging, consistent with our findings. Furthermore, we also strengthened this perspective by providing additional evidence of associations between LAI and frailty and multimorbidity, thereby offering a more comprehensive evaluation of its relevance to systemic aging. However, with regard to liver‐specific outcomes, only one study reported the predictive performance specifically for liver fibrosis and cirrhosis, and none conducted external validation for liver‐related outcomes. In this context, our studies advanced the field by employing a second‐generation modeling framework and providing a comprehensive, multicohort assessment of liver age's predictive performance for liver‐related outcomes across East Asian, European, and North American populations. These epidemiological findings were further supported by genetic evidence, which was helpful for elucidating the underlying biological mechanisms.

A novel finding of the current study is the pathogenesis of Alzheimer's disease associated with liver aging acceleration, as supported by evidence integrating genomics and proteomics. We first highlighted the involvement of a novel pathway—amyloid‐beta clearance in the enrichment analysis associated with genetically‐determined liver aging acceleration. We further observed strong positive associations between genetically‐determined liver aging acceleration and levels of amyloid precursor proteins (APP), a membrane protein central to the pathology of Alzheimer's disease. APP in physiological conditions follows a nonamyloidogenic pathway, but it can proceed to an amyloidogenic scenario, leading to the generation of extracellular deleterious Aβ plaques (O'brien and Wong 2011; Hardy and Higgins 1992). Intriguingly, we found an inverse association between genetically‐determined liver aging acceleration and circulating APP levels in CKB. This finding aligns with results from the UK Biobank (*n* = 49,912; 530 Alzheimer's disease cases), which also reported an inverse association between APP levels and Alzheimer's disease risk (Deng et al. 2025). Taken together, these lines of evidence suggested a potential link between liver aging acceleration and a higher risk of dementia. However, this finding should be interpreted with caution because the directionality and clinical implications remain to be established.

Although the LAI, unlike other biological aging models (e.g., liver dysregulation age, liver age scores, and liver biological age) (Ahadi et al. 2020; Nie et al. 2022; Oh et al. 2023), does not directly establish causality (Qiu et al. 2023), its consistent performance across independent cohorts and robust support from genetic evidence highlight its potential clinical utility. By incorporating both mortality and LRE information into the construction process (Nelson et al. 2020), the LAI more accurately captures liver‐specific biological aging and associated risks. This enhances its value as a risk stratification tool for systemic and liver‐specific aging. Given that its components are commonly measured in clinical practice, LAI may be readily incorporated into health examinations and primary care settings to identify individuals with accelerated liver aging who have not yet developed overt liver disease, thereby facilitating early risk stratification and targeted preventive strategies. In patients with established liver conditions, LAI may also complement existing clinical data to refine assessment of disease severity and guide follow‐up and intervention strategies. With further refinement and feature optimization, the LAI may support more precise and personalized strategies to promote healthy liver aging.

The main strengths of this study include the use of large‐scale prospective cohorts in Chinese adults, external validation in multiple cohorts, and adoption of mortality‐based methods (i.e., second‐generation). This study has several limitations. First, analysis of LRM was only conducted in CKB because of limited number of LRM cases in VCTE‐Prognosis cohort and the unavailability of detailed morality data in NHANES. However, we validated LRE as a liver‐related outcome in VCTE‐Prognosis cohort and showed consistent findings with CKB. Second, LAI was developed and validated on individuals aged 45 and above from the Chinese and US general population cohort, and a global multicenter MASLD cohort. The model is primarily applicable for liver aging prediction in middle‐aged and older populations. Third, the follow‐up period was relatively short in CKB and the number of deaths was also limited, which may partly explain the modest improvement in overall prediction error in our calibration analyses. However, we conducted Mendelian randomization with a median follow‐up of 3.53 years and confirmed the genetic associations of LAA with all‐cause mortality and liver‐related outcomes. Fourth, although we assessed the associations between LAI and frailty and multimorbidity, other dimensions of aging, such as disability and functional decline, as well as established BA clocks (e.g., DNA methylation clocks), were not included in the analysis due to a lack of available data. These data are expected to be collected in the upcoming 4th resurvey of the CKB study, which will enable more comprehensive assessment of LAI and further enhance its clinical and conceptual relevance. Fifth, Mendelian randomization was conducted only in East Asians because no genetic instruments of liver aging acceleration were available in Europeans. Future studies are warranted in European populations utilizing both LAI and genetic data to explore causality and biological mechanisms.

## 5 Conclusions

The current study showed the feasibility of constructing a noninvasive index for liver biological aging index across multicohorts. The LAI outperformed CA to predict all‐cause mortality and showed prospective associations with all‐cause mortality as well as liver‐related outcomes. Genetic analyses in East Asians suggested the causal associations between liver aging acceleration and liver‐related outcomes. Future studies with longer follow‐up are warranted to ascertain liver‐related mortality as well as to incorporate multiomics data (e.g., metabolomics, DNA methylations) to further elucidate biological mechanisms underlying liver aging. Additionally, research is warranted to determine whether modifying LAI through lifestyle or pharmacological interventions can translate into meaningful improvements in liver‐related outcomes.

## Author Contributions

Yuanjie Pang, Ming‐Hua Zheng, Vincent Wai‐Sun Wong, and Shanshan Wu contributed to the study concept and design; data acquisition involved all authors. Yuanjie Pang, Ming‐Hua Zheng, Vincent Wai‐Sun Wong, Zhiyu Wu, Shanshan Wu, Shuyao Song, and Xiao‐Dong Zhou performed the statistical analysis and drafted the manuscript. Liming Li, Zhengming Chen, and Junshi Chen: as the members of the CKB steering committee, designed and supervised the conduct of the whole study, obtained funding, and together with Jun Lv, Canqing Yu, Dianjianyi Sun, Yuting Han, Pei Pei, Ling Yang, Yiping Chen, Huaidong Du, Peng Liang, Geoffrey Ma, and Maxim Barnard, acquired the CKB data. All authors critically revised the manuscript for important intellectual content. Administrative, technical, or material support of the VCTE‐Prognosis cohort was provided by Xiao‐Dong Zhou, Ming‐Hua Zheng, and Vincent Wai‐Sun Wong. Supervision was provided by Yuanjie Pang, Ming‐Hua Zheng, and Vincent Wai‐Sun Wong. All authors approved the final version of the manuscript.

## Funding

This work was supported by the National Key R&D Program of China (2023YFC3606300), National Natural Science Foundation of China (82304223, 82192901, 82192904, 82192900, 82570631), and Beijing Nova Program (No. 20250484876 and No. 20230484349). The CKB baseline survey and the first resurvey were supported by a grant from the Kadoorie Charitable Foundation in Hong Kong. The long‐term follow‐up is supported by grants from the UK Wellcome Trust (212946/Z/18/Z, 202922/Z/16/Z, 104085/Z/14/Z, 088158/Z/09/Z), grants (2016YFC0900500) from the National Key R&D Program of China, National Natural Science Foundation of China (81390540, 91846303, 81941018), and the Chinese Ministry of Science and Technology (2011BAI09B01). The UK Medical Research Council (MC_UU_00017/1, MC_UU_12026/2, MC_U137686851), Cancer Research UK (C16077/A29186; C500/A16896), and the British Heart Foundation (CH/1996001/9454) provide core funding to the Clinical Trial Service Unit and Epidemiological Studies Unit at Oxford University for the project. The funders had no role in the study design, data collection, data analysis and interpretation, writing of the report, or the decision to submit the article for publication.

## Ethics Statement

This study was approved by the ethical committee and research council of the Chinese Center for Disease Control and Prevention (Beijing, China) and the Oxford Tropical Research Ethics Committee at the University of Oxford (Oxford, UK). All participants provided written informed consent.

## Conflicts of Interest

Dr. Tsochatzis reported receiving personal fees as an advisory board member for Boehringer, Novo Nordisk, Pfizer, and Siemens; receiving speaker fees from Echosens, Novo Nordisk, and AbbVie outside the submitted work. Dr. Boursier reported receiving grants and personal fees from Echosens outside the submitted work. Dr. Calleja reported receiving other from Echosens Clinical Trials during the conduct of the study; grants from Roche Pharma and other from Gilead Advisory Board outside the submitted work. Dr. Wah‐Kheong Chan reported serving as consultant or advisory board member for Zuellig Pharma, IPSEN, Kowa, Abbott, Roche, AbbVie, Boehringer Ingelheim, and Novo Nordisk; and a speaker for Novo Nordisk, Roche, Abbott, Echosens, Viatris, and Hisky Medical; and has received research grants from Abbott and Roche. Dr. Sanyal reported receiving grants from Intercept, personal consulting fees from Gilead, grants from Merck, personal consulting fees from Pfizer, grants and personal consulting fees from Eli Lilly, grants and personal consulting fees from Novo Nordisk, Boehringer Ingelheim, Novartis, Histoindex, and stock options from Genfit, Tiziana, Durect, Inversago, and personal consulting fees from Genentech, ALnylam, Regeneron, Zydus, LG chem, Hanmi, Madrigal, Path AI, 89 Bio, and stock options from Galmed outside the submitted work. Dr. De Lédinghen reported receiving nonfinancial support from Echosens during the conduct of the study. Dr. Newsome reported receiving grants from Novo Nordisk, advisory board and personal consulting fees, honoraria for lectures and travel expenses from Novo Nordisk, personal consulting and advisory board fees from Boehringer Ingelheim, Gilead, Intercept, Poxel Pharmaceuticals, Bristol‐Myers Squibb, Pfizer, MSD, Sun Pharma, Eli Lilly, Madrigal, GSK, and nonfinancial support for educational events from AiCME outside the submitted work. Dr. Castéra reported receiving personal fees for consulting and speakers bureau from Echosens during the conduct of the study; personal consultancy fees from Boston pharmaceutical and Gilead, speaker bureau and consultancy personal fees from GSK, personal speaker bureau fees from Inventiva, personal consultancy fees from Madrigal, personal Consultancy fees from MSD and Novo Nordisk, personal consultancy fees from Pfizer, Sagimet, and Siemens Healthineers outside the submitted work. Dr. Romero‐Gomez reported receiving personal fees from Echosens outside the submitted work. Dr. Kim reported personal fees from Gilead Sciences, personal fees from GSK, personal fees from Bayer, personal fees from Eisai, personal fees from AbbVie, personal fees from Echosens, personal fees from MSD, personal fees from Bristol‐Myers Squibb, and personal fees from AstraZeneca outside the submitted work, and grants from AbbVie, grants from Bristol‐Myers Squibb, and grants from Gilead Sciences outside the submitted work. Dr. Vincent Wai‐Sun Wong reported receiving personal speaker fees from Abbott, consultant and speaker fees from AbbVie, personal consultant fees from Boehringer Ingelheim, Echosens, Gilead Sciences, grants from Gilead Sciences, personal consultant fees from Intercept, Inventiva, Novo Nordisk, personal consultant fees from Pfizer, Sagimet Biosciences, TARGET PharmaSolutions, personal speaker fees from Unilab, personal consultant fees from Visirna, and being a cofounder of Illuminatio outside the submitted work. Dr. Zheng serves as a speaker for AstraZeneca, Hisky Medical Technologies, and Novo Nordisk; as a consultant for Boehringer Ingelheim and Eieling Technology; and has received consulting fees from Boehringer Ingelheim. No other disclosures were reported.

## Supporting information


**Data S1:** STROBE‐MR‐checklist‐fillable.

**Figure S1:** Correlation structure among the features for the LAI in the construction and validation cohorts.
**Figure S2:** Ridgeline plots of development and validation populations binned by decade for CA.
**Figure S3:** AUROC and Kaplan–Meier curve for LRE and LRM.
**Figure S4:** Associations of LAI with all‐cause mortality and liver‐related outcomes in CKB participants without and with MASLD.
**Table S1:** Transparent Reporting of a multivariable prediction model for Individual Prediction or Diagnosis (TRIPOD) guidelines.
**Table S2:** ICD‐10 codes used to define liver‐related events and mortality.
**Table S3:** Metrics of LAI in the development and validation cohorts.
**Table S4:** Colocalization analysis of LAI in Biobank Japan.
**Table S5:** Genetic associations of LAA with other diseases and biomarkers.
**Table S6:** Genetic associations of LAA with plasma proteins.
**Table S7:** Information of previous studies on Liver Biological Age.

## Data Availability

Details of how to access China Kadoorie Biobank data and details of the data release schedule are available from www.ckbiobank.org/site/Data+Access.

## References

[acel70565-bib-0002] Ahadi, S. , W. Zhou , S. M. Schüssler‐Fiorenza Rose , et al. 2020. “Personal Aging Markers and Ageotypes Revealed by Deep Longitudinal Profiling.” Nature Medicine 26, no. 1: 83–90. 10.1038/s41591-019-0719-5 PMC7301912 31932806

[acel70565-bib-0003] Chen, L. , Q. Rao , M. Gao , G. Lv , and F. Tacke . 2025. “Prospects of Mendelian Randomization in Hepatology: A Comprehensive Literature Review With Practice Guidance.” Clinical and Molecular Hepatology 31, no. 4: 1115–1138. 40485101 10.3350/cmh.2025.0541 PMC12538149

[acel70565-bib-0004] Chen, Z. , J. Chen , R. Collins , et al. 2011. “China Kadoorie Biobank of 0.5 Million People: Survey Methods, Baseline Characteristics and Long‐Term Follow‐Up.” International Journal of Epidemiology 40, no. 6: 1652–1666. 22158673 10.1093/ije/dyr120 PMC3235021

[acel70565-bib-0005] Collins, G. S. , J. B. Reitsma , D. G. Altman , et al. 2015. “Transparent Reporting of a Multivariable Prediction Model for Individual Prognosis or Diagnosis (TRIPOD): The TRIPOD Statement.” BMC Medicine 13, no. 1: g7594. 10.1186/s12916-014-0241-z PMC4284921 25563062

[acel70565-bib-0006] Deng, Y. T. , J. You , Y. He , et al. 2025. “Atlas of the Plasma Proteome in Health and Disease in 53,026 Adults.” Cell 188, no. 1: 253–271.e7. 39579765 10.1016/j.cell.2024.10.045

[acel70565-bib-0007] Fong, S. , K. Pabis , D. Latumalea , et al. 2024. “Principal Component‐Based Clinical Aging Clocks Identify Signatures of Healthy Aging and Targets for Clinical Intervention.” Nature Aging 4, no. 8: 1137–1152. 38898237 10.1038/s43587-024-00646-8 PMC11333290

[acel70565-bib-0008] Goeminne, L. J. E. , A. Vladimirova , A. Eames , et al. 2025. “Plasma Protein‐Based Organ‐Specific Aging and Mortality Models Unveil Diseases as Accelerated Aging of Organismal Systems.” Cell Metabolism 37, no. 1: 205–222.e6. 39488213 10.1016/j.cmet.2024.10.005

[acel70565-bib-0009] Hardy, J. A. , and G. A. Higgins . 1992. “Alzheimer's Disease: The Amyloid Cascade Hypothesis.” Science 256, no. 5054: 184–185. 1566067 10.1126/science.1566067

[acel70565-bib-0001] IHME . 2025. GBD Results [M]. IHME, University of Washington.

[acel70565-bib-0010] Im, P. K. , N. Wright , L. Yang , et al. 2023. “Alcohol Consumption and Risks of More Than 200 Diseases in Chinese Men.” Nature Medicine 29, no. 6: 1476–1486. 10.1038/s41591-023-02383-8 PMC10287564 37291211

[acel70565-bib-0011] Le Goallec, A. , S. Diai , S. Collin , et al. 2022. “Using Deep Learning to Predict Abdominal Age From Liver and Pancreas Magnetic Resonance Images.” Nature Communications 13, no. 1: 1979. 10.1038/s41467-022-29525-9 PMC9007982 35418184

[acel70565-bib-0012] Levine, M. E. , A. T. Lu , A. Quach , et al. 2018. “An Epigenetic Biomarker of Aging for Lifespan and Healthspan.” Aging 10, no. 4: 573–591. 29676998 10.18632/aging.101414 PMC5940111

[acel70565-bib-0013] Lin, H. , H. W. Lee , T. C. Yip , et al. 2024. “Vibration‐Controlled Transient Elastography Scores to Predict Liver‐Related Events in Steatotic Liver Disease.” JAMA 331, no. 15: 1287–1297. 38512249 10.1001/jama.2024.1447 PMC10958386

[acel70565-bib-0014] Man, S. , Y. Deng , Y. Ma , et al. 2023. “Prevalence of Liver Steatosis and Fibrosis in the General Population and Various High‐Risk Populations: A Nationwide Study With 5.7 Million Adults in China.” Gastroenterology 165, no. 4: 1025–1040. 37380136 10.1053/j.gastro.2023.05.053

[acel70565-bib-0015] Moqri, M. , C. Herzog , J. R. Poganik , et al. 2024. “Validation of Biomarkers of Aging.” Nature Medicine 30, no. 2: 360–372. 10.1038/s41591-023-02784-9 PMC11090477 38355974

[acel70565-bib-0016] Nelson, P. G. , D. E. L. Promislow , and J. Masel . 2020. “Biomarkers for Aging Identified in Cross‐Sectional Studies Tend to Be Non‐Causative.” Journals of Gerontology. Series A, Biological Sciences and Medical Sciences 75, no. 3: 466–472. 31353411 10.1093/gerona/glz174 PMC7457180

[acel70565-bib-0017] Nie, C. , Y. Li , R. Li , et al. 2022. “Distinct Biological Ages of Organs and Systems Identified From a Multi‐Omics Study.” Cell Reports 38, no. 10: 110459. 35263580 10.1016/j.celrep.2022.110459

[acel70565-bib-0018] O'brien, R. J. , and P. C. Wong . 2011. “Amyloid Precursor Protein Processing and Alzheimer's Disease.” Annual Review of Neuroscience 34: 185–204. 10.1146/annurev-neuro-061010-113613 PMC3174086 21456963

[acel70565-bib-0019] Oh, H. S. , J. Rutledge , D. Nachun , et al. 2023. “Organ Aging Signatures in the Plasma Proteome Track Health and Disease.” Nature 624, no. 7990: 164–172. 38057571 10.1038/s41586-023-06802-1 PMC10700136

[acel70565-bib-0020] Pang, Y. , C. Kartsonaki , I. Turnbull , et al. 2018. “Diabetes, Plasma Glucose, and Incidence of Fatty Liver, Cirrhosis, and Liver Cancer: A Prospective Study of 0.5 Million People.” Hepatology 68, no. 4: 1308–1318. 29734463 10.1002/hep.30083 PMC6220764

[acel70565-bib-0021] Qiu, W. , H. Chen , M. Kaeberlein , and S. I. Lee . 2023. “ExplaiNAble BioLogical Age (ENABL Age): An Artificial Intelligence Framework for Interpretable Biological Age.” Lancet Healthy Longevity 4, no. 12: e711–e723. 37944549 10.1016/S2666-7568(23)00189-7

[acel70565-bib-0022] Sehgal, R. , Y. Markov , C. Qin , et al. 2025. “Systems Age: A Single Blood Methylation Test to Quantify Aging Heterogeneity Across 11 Physiological Systems.” Nature Aging 5, no. 9: 1880–1896. 40954326 10.1038/s43587-025-00958-3 PMC13222069

[acel70565-bib-0023] Song, S. , Z. Wu , J. Lv , et al. 2024. “Dietary Factors and Patterns in Relation to Risk of Later‐Onset Ulcerative Colitis in Chinese: A Prospective Study of 0.5 Million People.” Alimentary Pharmacology & Therapeutics 59, no. 11: 1425–1434. 38654428 10.1111/apt.17963

[acel70565-bib-0024] Tian, Y. E. , V. Cropley , A. B. Maier , N. T. Lautenschlager , M. Breakspear , and A. Zalesky . 2023. “Heterogeneous Aging Across Multiple Organ Systems and Prediction of Chronic Disease and Mortality.” Nature Medicine 29, no. 5: 1221–1231. 10.1038/s41591-023-02296-6 37024597

[acel70565-bib-0025] Xing, W. , W. Gao , Z. Zhao , et al. 2023. “Dietary Flavonoids Intake Contributes to Delay Biological Aging Process: Analysis From NHANES Dataset.” Journal of Translational Medicine 21, no. 1: 492. 37480074 10.1186/s12967-023-04321-1 PMC10362762

[acel70565-bib-0026] Yang, G. , C. Rao , J. Ma , et al. 2006. “Validation of Verbal Autopsy Procedures for Adult Deaths in China.” International Journal of Epidemiology 35, no. 3: 741–748. 16144861 10.1093/ije/dyi181

[acel70565-bib-0027] Zhong, L. , L. Wang , J. N. Syed , J. Yang , and Y. Zhang . 2025. “Liver Aging: Underlying Mechanisms and Therapeutic Strategies.” Molecular Aspects of Medicine 105: 101397. 40886472 10.1016/j.mam.2025.101397

[acel70565-bib-0028] Zhou, X. D. , S. U. Kim , T. C. Yip , et al. 2024. “Long‐Term Liver‐Related Outcomes and Liver Stiffness Progression of Statin Usage in Steatotic Liver Disease.” Gut 73, no. 11: 1883–1892. 39089860 10.1136/gutjnl-2024-333074

