# Supplementary material for: Liver Aging Index: A Noninvasive Score for Liver Biological Aging and Liver‐Related Outcomes in Multicohorts
Source: Aging Cell. 2026 May 31;25(6):e70565. doi: 10.1111/acel.70565 (PMC13239111; doi:10.1111/acel.70565)
Supplement: Supplementary file 2 — Figure S1: Correlation structure among the features for the LAI in the construction and validation cohorts. Figure S2: Ridgeline plots of development and validation populations binned by decade for CA. Figure S3: AUROC and Kaplan–Meier curve for LRE and LRM. Figure S4: Associations of LAI with all‐cause mortality and liver‐related outcomes in CKB participants without and with MASLD. Table S1: Transparent Reporting of a multivariable prediction model for Individual Prediction or Diagnosis (TRIPOD) guidelines. Table S2: ICD‐10 codes used to define liver‐related events and mortality. Table S3: Metrics of LAI in the development and validation cohorts. Table S4: Colocalization analysis of LAI in Biobank Japan. Table S5: Genetic associations of LAA with other diseases and biomarkers. Table S6: Genetic associations of LAA with plasma proteins. Table S7: Information of previous studies on Liver Biological Age. [file ACEL-25-e70565-s002.zip › acel70565-sup-0002-TableS1-S87-FigureS1-S5@2026-04-27 Supplementary Materials - tracked.docx]

**Supplemental Digital Content**

**Liver Aging Index: a non-invasive score for liver biological aging and liver-related outcomes in multi-cohorts**

Running title: Liver aging index for liver-related outcomes

Zhiyu Wu^1^*, Shanshan Wu^2^*, Shuyao Song^1^*, Yating Huang^1^, Canqing Yu^1,3,4^, Dianjianyi Sun^1,3,4^, Pei Pei^1,3^, Ling Yang^5^, Yiping Chen^5^, Huaidong Du^5^, Robin Walters^5^, Iona Millwood^5^, Hao Xu^6^, Xiaoming Yang^5^, Junshi Chen^7^, Seung Up Kim^8^, Salvatore Petta^9^, Atsushi Nakajima^10^, Emmanuel Tsochatzis^11^, Jérôme Boursier^12^, Elisabetta Bugianesi^13^, Wah-Kheong Chan^14^, Manuel Romero-Gomez^15^, José Luis Calleja^16^, Victor de Lédinghen^17^, Laurent Castéra^18^, Arun J. Sanyal^19^, George Boon-Bee Goh^20^, Philip Noel Newsome^21^, Jian-Gao Fan^22^, Michelle Lai^23^, Xiao-Dong Zhou^24^, Zhengming Chen^5^, Jun Lv^1,3,4,25^, Liming Li^1,3,4^, Vincent Wai-Sun Wong^26,27†^, Ming-Hua Zheng^24,28,29†^, Yuanjie Pang^1,3,4†^ on behalf of the China Kadoorie Biobank Collaborative Group and VCTE-Prognosis Study Group

*Joint first authors ^†^Joint corresponding authors

1. Department of Epidemiology & Biostatistics, School of Public Health, Peking University, Beijing 100191, China
2. Department of Gastroenterology, Beijing Friendship Hospital, Capital Medical University, State Key Laboratory of Digestive Health, National Clinical Research Center for Digestive Diseases, Beijing 100050, China
3. Peking University Center for Public Health and Epidemic Preparedness & Response, Beijing 100191, China
4. Key Laboratory of Epidemiology of Major Diseases (Peking University), Ministry of Education
5. Clinical Trial Service Unit & Epidemiological Studies Unit (CTSU), Nuffield Department of Population Health, University of Oxford, United Kingdom
6. Tongxiang Center for Disease Control and Prevention, Tongxiang, Zhejiang 314500, China
7. China National Center for Food Safety Risk Assessment, Beijing 100022, China
8. Department of Internal Medicine, Yonsei University College of Medicine, Seoul, Republic of Korea
9. Sezione di Gastroenterologia, Di.Bi.M.I.S., University of Palermo, Italy
10. Department of Gastroenterology and Hepatology, Yokohama City University Graduate School of Medicine, Yokohama, Japan
11. University College London Institute for Liver and Digestive Health, Royal Free Hospital, London, United Kingdom
12. Hepato-Gastroenterology and Digestive Oncology Department, Angers University Hospital, Angers, France
13. Department of Medical Sciences, Division of Gastroenterology and Hepatology, A.O. Città della Salute e della Scienza di Torino, University of Turin, Turin, Italy
14. Gastroenterology and Hepatology Unit, Department of Medicine, Faculty of Medicine, University of Malaya, Malaysia
15. UCM Digestive Diseases, Virgen del Rocío University Hospital, Centro de Investigación Biomédica en Red en Enfermedades Hepáticas y Digestivas (CIBEREHD), Institute of Biomedicine of Seville (IBiS), University of Seville, Spain
16. Department of Gastroenterology and Hepatology, Hospital Universitario Puerta de Hierro Majadahonda, Madrid, Spain
17. Echosens, Paris, France
18. Université Paris Cité, UMR1149 (CRI), INSERM, Paris, France; Service d'Hépatologie, Hôpital Beaujon, Assistance Publique-Hôpitaux de Paris (AP-HP), Clichy, France
19. Division of Gastroenterology, Hepatology and Nutrition, Department of Internal Medicine, Virginia Commonwealth University School of Medicine, Richmond, VA, USA
20. Department of Gastroenterology and Hepatology, Singapore General Hospital, Singapore
21. Institute of Hepatology, Faculty of Life Sciences & Medicine, King’s College London and King’s College Hospital, London, UK
22. Department of Gastroenterology and Hepatology, School of Medicine, Shanghai Jiao Tong University, Shanghai, China
23. Division of Gastroenterology & Hepatology, Beth Israel Deaconess Medical Center, Harvard Medical School, Boston, MA, USA
24. MAFLD Research Center, Department of Hepatology, the First Affiliated Hospital of Wenzhou Medical University, Wenzhou, China
25. State Key Laboratory of Vascular Homeostasis and Remodeling, Peking University, Beijing 100191, China
26. Medical Data Analytics Centre, Department of Medicine and Therapeutics, The Chinese University of Hong Kong, Hong Kong, China
27. State Key Laboratory of Digestive Disease, Institute of Digestive Disease, The Chinese University of Hong Kong, Hong Kong, China
28. Institute of Hepatology, Wenzhou Medical University, Wenzhou, China
29. Key Laboratory of Diagnosis and Treatment for the Development of Chronic Liver Disease in Zhejiang Province, Wenzhou, China

**Address for correspondence:**

| Dr. Yuanjie Pang | Prof. Ming-Hua Zheng | Prof. Vincent Wai-Sun Wong |
| --- | --- | --- |
| Department of Epidemiology & Biostatistics,  School of Public Health,  Peking University | MAFLD Research Center,  Department of Hepatology,  The First Affiliated Hospital of Wenzhou Medical University | Medical Data Analytics Centre,  Department of Medicine and Therapeutics,  The Chinese University of Hong Kong |
| Beijing, China | Wenzhou, China | Hong Kong, China |
| ypang@bjmu.edu.cn | zhengmh@wmu.edu.cn | wongv@mect.cuhk.edu.hk |

Revised in April 2026

**Table of content**

[Members of the China Kadoorie Biobank collaborative group 4](#_Toc228179379)

[Members of the VCTE-Prognosis Study Group 5](#_Toc228179380)

[Supplementary Methods 6](#_Toc228179381)

[Supplementary Table 1. Transparent Reporting of a multivariable prediction model for Individual Prediction or Diagnosis (TRIPOD) guidelines 78](#_Toc228179382)

[Supplementary Table 2. ICD-10 codes used to define liver-related events and mortality 80](#_Toc228179383)

[Supplementary Table 3. Metrics of LAI in the development and validation cohorts 81](#_Toc228179384)

[Supplementary Table 4. Calibration results of LAI for liver-related events 82](#_Toc228179385)

[Supplementary Table 5. Colocalization analysis of LAI in Biobank Japan 83](#_Toc228179386)

[Supplementary Table 6. Genetic associations of LAA with other diseases and biomarkers 84](#_Toc228179387)

[Supplementary Table 7. Genetic associations of LAA with plasma proteins 85](#_Toc228179388)

[Supplementary Table 8. Information of previous studies on Liver Biological Age 86](#_Toc228179389)

[Supplementary Figure 1. Correlation structure among the features for the LAI in the construction and validation cohorts 88](#_Toc228179390)

[Supplementary Figure 2. Ridgeline plots of development and validation populations binned by decade for CA 89](#_Toc228179391)

[Supplementary Figure 3. Model performance of LAI for liver-related outcome 90](#_Toc228179392)

[Supplementary Figure 4. Associations of LAI with all-cause mortality and liver-related outcomes in CKB participants without and with MASLD 92](#_Toc228179393)

[Supplementary Figure 5. Associations of LAI and LAA with geriatric indicators in CKB 93](#_Toc228179394)

# Members of the China Kadoorie Biobank collaborative group

International Steering Committee: Junshi Chen, Zhengming Chen (PI), Robert Clarke, Rory Collins, Yu Guo, Liming Li (PI), Jun Lv, Richard Peto, Robin Walters. International Co-ordinating Centre, Oxford: Daniel Avery, Derrick Bennett, Ruth Boxall, Sue Burgess, Ka Hung Chan, Yumei Chang, Yiping Chen, Zhengming Chen, Johnathan Clarke, Robert Clarke, Huaidong Du, Ahmed Edris Mohamed, Zammy Fairhurst-Hunter, Hannah Fry, Simon Gilbert, Alex Hacker, Mike Hill, Michael Holmes, Pek Kei Im, Andri Iona, Maria Kakkoura, Christiana Kartsonaki, Rene Kerosi, Kuang Lin, Mohsen Mazidi, Iona Millwood, Sam Morris, Qunhua Nie, Alfred Pozarickij, Paul Ryder, Saredo Said, Sam Sansome, Dan Schmidt, Paul Sherliker, Rajani Sohoni, Becky Stevens, Iain Turnbull, Robin Walters, Lin Wang, Neil Wright, Ling Yang, Xiaoming Yang, Pang Yao.

National Co-ordinating Centre, Beijing: Yu Guo, Xiao Han, Can Hou, Jun Lv, Pei Pei, Chao Liu, Canqing Yu, Qingmei Xia. 10 Regional Co-ordinating Centres: Qingdao CDC: Zengchang Pang, Ruqin Gao, Shanpeng Li, Haiping Duan, Shaojie Wang, Yongmei Liu, Ranran Du, Yajing Zang, Liang Cheng, Xiaocao Tian, Hua Zhang, Yaoming Zhai, Feng Ning, Xiaohui Sun, Feifei Li. Licang CDC: Silu Lv, Junzheng Wang, Wei Hou. Heilongjiang Provincial CDC: Wei Sun, Shichun Yan, Xiaoming Cui. Nangang CDC: Chi Wang, Zhenyuan Wu, Yanjie Li, Quan Kang. Hainan Provincial CDC: Huiming Luo, Tingting Ou. Meilan CDC: Xiangyang Zheng, Zhendong Guo, Shukuan Wu, Yilei Li, Huimei Li. Jiangsu Provincial CDC: Ming Wu, Yonglin Zhou, Jinyi Zhou, Ran Tao, Jie Yang, Jian Su. Suzhou CDC: Fang Liu, Jun Zhang, Yihe Hu, Yan Lu, Liangcai Ma, Aiyu Tang, Shuo Zhang, Jianrong Jin, Jingchao Liu. Guangxi Provincial CDC: Mei Lin, Zhenzhen Lu. Liuzhou CDC: Lifang Zhou, Changping Xie, Jian Lan, Tingping Zhu, Yun Liu, Liuping Wei, Liyuan Zhou, Ningyu Chen, Yulu Qin, Sisi Wang. Sichuan Provincial CDC: Xianping Wu, Ningmei Zhang, Xiaofang Chen, Xiaoyu Chang. Pengzhou CDC: Mingqiang Yuan, Xia Wu, Xiaofang Chen, Wei Jiang, Jiaqiu Liu, Qiang Sun. Gansu Provincial CDC: Faqing Chen, Xiaolan Ren, Caixia Dong. Maiji CDC: Hui Zhang, Enke Mao, Xiaoping Wang, Tao Wang, Xi zhang. Henan Provincial CDC: Kai Kang, Shixian Feng, Huizi Tian, Lei Fan. Huixian CDC: XiaoLin Li, Huarong Sun, Pan He, Xukui Zhang. Zhejiang Provincial CDC: Min Yu, Ruying Hu, Hao Wang. Tongxiang CDC: Xiaoyi Zhang, Yuan Cao, Kaixu Xie, Lingli Chen, Dun Shen. Hunan Provincial CDC: Xiaojun Li, Donghui Jin, Li Yin, Huilin Liu, Zhongxi Fu. Liuyang CDC: Xin Xu, Hao Zhang, Jianwei Chen, Yuan Peng, Libo Zhang, Chan Qu.

# Members of the VCTE-Prognosis Study Group

The VCTE-Prognosis Study Group consists of the following authors: Seung Up Kim, Terry Cheuk-Fung Yip, Salvatore Petta, Atsushi Nakajima, Emmanuel Tsochatzis, Jérôme Boursier, Elisabetta Bugianesi, Hannes Hagstrom, Wah-Kheong Chan, Manuel Romero-Gómez, José Luis Calleja, Victor de Lédinghen, Laurent Castéra, Arun J. Sanyal, George Boon-Bee Goh, Philip Noel Newsome, Jiangao Fan, Michele Lai, Céline Fournier-Poizat, Hye Won Lee, Grace Lai-Hung Wong, Angelo Armandi, Ying Shang, Grazia Pennisi, Elba Llop, Masato Yoneda, Mirko Zoncapè, Marc Saint-Loup, Rocio Gallego-Durán, Paloma Fernández, Amon Asgharpour, Huapeng Lin, Wen-Yue Liu, Xiao-Dong Zhou, Kevin Kim-Jun Teh, Mandy Sau-Wai Chan, Ming-Hua Zheng, Vincent Wai-Sun Wong

# Supplementary Methods

**1 Data collection and processing**

**1.1 Study population**

*The China Kadoorie Biobank (CKB)*

The CKB study is a nationwide population-based prospective cohort study. Details of the study design have been described elsewhere.^1^ In brief, 512,724 participants aged 30-79 years were recruited from five urban and five rural areas in the 2004-2008 baseline survey. Three periodic resurveys were conducted in a sample of about 5% of surviving participants. The third resurvey took place between 2020 and 2021, involving 25,087 participants.^2^ In the CKB 3rd resurvey (n=25,087), after excluding (1) participants with missing liver aging index (LAI) features (n=3,156); (2) participants with a prior diagnosis of cancer (n=92), cirrhosis or chronic hepatitis (n=201), or missing baseline medical history (n=9), 21,629 participants were included.

*The National Health and Nutrition Examination Survey (NHANES)*

The NHANES from the US National Center for Health Statistics (NCHS) conducts interviews and physical examinations to assess the health and nutrition data for all ages in the United States. Since 1999, data have been collected and released at 2-year intervals. For this study, we include NHANES data collected between 2017 and 2018 (n=9,254). In our analysis, we excluded (1) 3,398 participants with missing LAI features, (2) 75 participants with liver cancer (n=3) and cirrhosis or chronic hepatitis (n=72), and 2369 participants aged < 45 years. Hence, a total of 3,412 participants aged ≥45 years were included in the analysis.

*The VCTE-Prognosis Study*

The VCTE-Prognosis Study included data from a natural history cohort of 18,057 patients with clinically diagnosed MASLD who underwent VCTE at tertiary referral centers across the United States, Europe, and Asia. For this study, we excluded (1) participants <45 years (n=5,092) or with missing age (n =115), as well as (2) missing mortality information (n=680), leaving 12,170 participants aged ≥45 years for the final analysis.

**1.2 Candidate predictors**

In CKB 3rd resurvey dataset, height and weight were recorded by trained technicians using calibrated instruments with standard protocols. Standing height and body weight were measured by a height meter and a body fat meter (Tanita BC418MA, ‎TANITA; Japan), respectively. Body mass index was calculated as weight in kilograms divided by height in meters squared. Vibration-controlled transient elastography (VCTE) was performed by FibroTouch FT-100 (Hisky, Wuxi, China) to measure the fat attenuation parameter (FAP) and liver stiffness measurement (LSM) to assess liver steatosis and fibrosis, respectively. FibroTouch is widely adopted in Chinese healthcare institutions due to its cost-effectiveness, accessibility, and operational simplicity, and it has shown comparable diagnostic accuracy to FibroScan for grading fibrosis^4–6^. For each participant, a 10 mL blood sample was collected without a fasting requirement, but with the time since the last meal being recorded. Random plasma glucose (RPG) was tested on-site using SureStep Plus meters (Lifescan, Johnson & Johnson). For clinical biochemistry tests, 4 ml blood samples were collected and centrifuged in the field, kept refrigerated at 4°C, and tested within 24 hours. Total cholesterol (TC), triglyceride, and high-density lipoprotein cholesterol (HDL-C) were assayed using the BS-240 clinical chemical analyzer (Mindray, China). Low-density lipoprotein cholesterol (LDL-C) was estimated based on TC, HDL-C, and triglycerides using the Friedewald formula ^4^. Alanine Aminotransferase (ALT), Aspartate Aminotransferase (AST), and Gamma-Glutamyl Transferase (GGT) were measured using the BS-240 Clinical Chemistry Analyzer (Mindray, China). All assays were performed according to the manufacturer’s specifications, utilizing standardized reagents, calibrators, and settings.

In the NHANES 2017-2018 dataset, the body measures data and all BP determinations (systolic and diastolic) were collected in the Mobile Examination Center (MEC) by trained health technicians. The health technician was assisted by a recorder during the examination ^7^. The participant’s age at the time of the screening interview determined the body measures examination protocol. Liver stiffness was measured using the FibroScan model 502 V2 Touch (Echosens, Waltham, MA) ^8^. The device also simultaneously measures the ultrasound attenuation related to the presence of hepatic steatosis and records the controlled attenuation parameter (CAPTM) as an indicator of fat content in the liver. Serum specimens are processed, stored, and shipped to the University of Minnesota-Advanced Research Diagnostics Laboratory (ARDL), Minneapolis, MN for analysis. Vials are stored under appropriate refrigerated (2-8°C) conditions until they are shipped to Collaborative Laboratory Services for testing^7^. ALT, AST and GGT were measured using an automated clinical chemistry analyzer, the Roche Cobas 6000 system, module c501 ^9^.

As for VCTE-Prognosis cohort, FAP and LSM were measured by experienced operators who had received formal training from the manufacturer or certified delegates using the VCTE machine (FibroScan, Echosens, Paris, France) within one month of the clinic visit and blood tests ^10–12^. Eligibility required a diagnosis of MASLD through liver histology (steatosis in ≥5% of hepatocytes) or imaging methods (ultrasonography, computed tomography, magnetic resonance imaging, or controlled attenuation parameter by VCTE). At each clinic visit, the medical history of a patient was recorded. A venous blood sample was taken after at least 8 hours of fasting for liver biochemistry and complete blood cell count.

In VCTE-Prognosis cohort and NHANES datasets, we performed missing data imputation separately using the R package missRanger^13^, which combined random forest imputation with predictive mean matching. We set the maximum number of trees for the random forest to 200, but left all other random forest hyperparameters at their default. The variables used as candidate predictors in the imputation included all baseline, non-nested variables, the Nelson-Aalen estimate of cumulative mortality hazard, and the all-cause mortality event indicator.

**1.3 Outcomes and chronological age**

In CKB, the vital status of each participant was determined periodically through the China CDC's Disease Surveillance Points (DSP) system and the national health insurance system, supplemented by regular checks against local residential and health insurance records and by annual active confirmation through street committees or village administrators.^14^ In addition, information about the occurrence of major diseases and any episodes of hospitalization was collected through linkages, using each participant's unique national identification number, with disease registries and national health insurance claim databases, which have almost universal coverage in the study areas. All events were coded using the International Classification of Diseases, 10th revision (ICD-10), by trained staff who were blinded to baseline information and reviewed centrally for consistency.^15^ Mortality data were accessed from the CKB data with a censoring date of 31 December 2024 for all participants. LREs were defined by major clinical complications of cirrhosis and advanced chronic liver diseases: 1) hepatocarcinogenesis: hepatocellular carcinoma (HCC; C22.0, C22.7); 2) portal hypertension-related event: variceal bleeding (I85.0, I85.9, I98.2, I98.3) and ascites (K76.6, K76.7, K76.8, K76.9); 3) liver dysfunction-related events: hepatic failure/acute-on-chronic liver failure (K72.0, K72.1, K72.9), overt hepatic encephalopathy, and other severe conditions. A detailed list of ICD-10 codes can be found in **Table S2**. Corresponding ICD-10 codes were also used to define LRM. In CKB, MASLD was diagnosed based on liver steatosis (FAP≥ 244 Db/m) and the presence of any of the five categories of cardiometabolic ^16^. In CKB, age at study date wea calculated from date of birth and study date.

For NHANES 2017-2018, public-use Linked Mortality Files (LMF) provide all-cause mortality follow-up from survey participation through December 31, 2019, for adult participants^17^. In the VCTE-Prognosis cohort, the outcomes of interest included incident all-cause mortality and liver-related events (LRE). The outcome was a composite endpoint of LRE including hepatocellular carcinoma , hepatic decompensation (ascites, variceal hemorrhage, hepatic encephalopathy, or hepatorenal syndrome), liver transplant, and liver-related death^12^.The diagnosis of the events was based on prospective follow up, medical record review, or validated registries with positive predictive values of at least 90%^10–12^.

**1.4 Ethnic statements**

For CKB, the Ethical Review Committee of the Chinese Center for Disease Control and Prevention (Beijing, China) and the Oxford Tropical Research Ethics Committee, University of Oxford (UK), approved the study. For NHANES, the National Centre for Health Statistics Research Ethics Review Board approved all NHANES protocols. For the VCTE-Prognosis cohort study, the study protocol was approved by the institutional review boards of the participating sites. The study was conducted in accordance with the principles of the Declaration of Helsinki. The patients provided informed written consent for the prospective programs at the local sites.

**2 Liver aging index approach**

Liver aging index was constructed using a two-step modeling approach with the CKB as the training dataset, and internal validation by 10-fold validation. External validation was subsequently conducted in the VCTE-Prognosis cohort and the NHANES cohort. All analyses were stratified by sex. We adopt the framework of ‘Cox-Gompertz’ biological age, following the approach by Levine et al and Fong et al^18,19^.


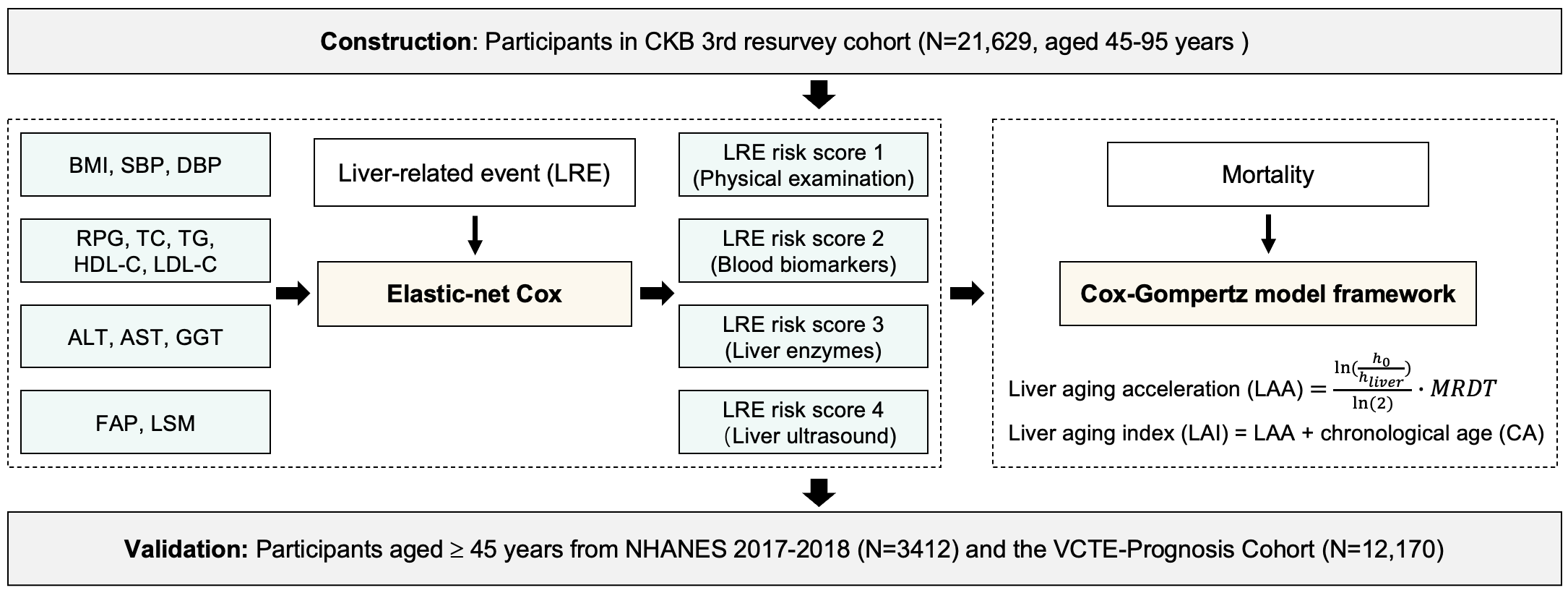


**Figure A. The workflow of the liver aging index construction and validation**

$h_{0}$: mortality hazard from null Cox model (CA only); $MRDT$: mortality rate doubling time by CA; $h_{liver}$:mortality hazard from liver Cox model (CA + LRE risk scores).

Abbreviation: RPG, random plasma glucose; TC, total cholesterol; TG, triglycerides; HDL-C; high-density lipoprotein cholesterol; LDL-C, low-density lipoprotein cholesterol; FAP, fat attenuation parameter; LSM, liver stiffness measurement; BMI，body mass index；SBP, systolic blood pressure; DBP, diastolic blood pressure.

**2.1** **Step 1: Risk prediction based on elastic-net Cox regression**

We grouped the 13 biomarkers into 4 categories based on their measurement type and liver aging biomarkers framework by the Aging Biomarker Consortium^20^: liver enzymes, blood biomarkers, liver imaging markers, and body measurement biomarkers. Elastic-net Cox models were then applied to predict LRE risk for each group. The models were trained on the CKB dataset (n = 21,629) using a 10-fold cross-validation approach. The regularization parameter (lambda.min) was selected to minimize cross-validation error, with **alpha set to 0** to implement a ridge regression framework, ensuring all biomarkers were retained. Model performance was evaluated using the concordance index (C-index), and linear predictors (type="link") generated from the optimized models were used as features for predicting mortality risk in Step 2.

**2.2 Step 2: the LAI Cox-Gompertz framework**

We employed a **Cox-Gompertz framework** to construct the Liver Aging Index (LAI) based on second-generation biological age clocks, following Levine et al. and Fong et al.^18,19^ Cox proportional hazards models were developed in the CKB 3rd resurvey training cohort, and the derived parameters were applied to validate LAI in the NHANES 2017-2018 and VCTE-Prognosis cohorts.

We first generated two Cox proportional hazard models for all-cause mortality in the training cohort, the CKB 3rd resurvey dataset.

(A) The null model predicted mortality hazard (h₀) based on chronological age (CA) alone, yielding sex-specific mortality rate doubling time (MRDTₛₑₓ).

$$h_{i}^{null}\left( t \right)=h_{0}^{null}\left( t \right)*\exp(\beta_{CA}^{null}*CA)$$

(B) The liver model was a second Cox model constructed by considering the liver-related biomarkers for each subject of the training cohort. The final model was then used to predict the hazard of dying as a function of an individual’s liver function (h_liver_).

$$h_{i}\left( t \right)=h_{0}\left( t \right)*\exp(\beta_{CA}*CA+\beta_{1}*X_{1}+\beta_{2}*X_{2}+\cdots\beta_{m}*X_{m})$$

(C) Finally, differences in ‘Gompertz age’ are calculated that result in an equivalent relative hazard ratio h_liver_/h_0_, thereby converting the hazard ratio into a corrected ‘Gompertz age’ (Δage) as liver aging acceleration follows:

$$Liver aging acceleration \left( LAA \right)=\Delta age=\frac{ln(\frac{h_{\mathrm{liver}}}{h_{0}})}{ln(2)}\cdot{MRDT}_{sex}$$

(D) The final LAI was then calculated by adding this age correction to the subject actual CA: Subjects were further classified into ‘liver aging acceleration’ and ‘liver aging deceleration’ populations based on LAA quartiles, with the highest quartile representing accelerated liver aging (LAI high) and the lowest quartile representing decelerated liver aging (LAI low).

$$Liver aging index (LAI)=CA+LAA$$

The **LAI-13 calculator** is available on <https://liveragingindex.github.io> for replication.

**2.3 LAI-5: the simplified version of Liver Aging Index**

To enhance clinical translatability by focusing on core liver-related biomarkers, we further developed a simplified subset of the Liver Aging Index, termed **LAI-5**, which exclusively incorporates five key liver function and structural markers: FAP, LSM, ALT, AST, and GGT.

Cox model for LAI-5:

$$h_{i}\left( t \right)=h_{0}\left( t \right)*\exp(\beta_{CA}*CA+\beta_{1}*FAP+\beta_{2}*LSM+\cdots\beta_{5}*GGT)$$

The NULL model based on CA alone was used to calculate relative hazard ratios and ‘Gompertz age’ (LAA -5) as follows:

LAA -5$=\frac{\ln(\frac{h}{h_{null}})}{\ln(2)}\cdot MRDT$, LAA -5$=LAA+CA$

**Table A. LAI-5 parameters**

| **Sex** | **Variable** | **β** |
| --- | --- | --- |
| Men | CA | 0.1151 |
|  | FAP | -0.1788 |
|  | LSM | 0.14474 |
|  | ALT | -0.0630 |
|  | AST | -0.0937 |
|  | GGT | 0.3257 |
|  | Null model | 0.1169 |
|  | MRDT | 6.0200 |
| Women | CA | 0.1236 |
|  | FAP | -0.0816 |
|  | LSM | 0.1109 |
|  | ALT | -0.1071 |
|  | AST | -0.0495 |
|  | GGT | 0.2708 |
|  | Null model | 0.1270 |
|  | MRDT | 5.6100 |

**3. Geriatric indicators**

**3.1 Frailty**

We constructed the frailty index following a previously validated approach in the CKB ^21^. Briefly, candidate deficits were identified from self-reported and measured variables collected at baseline and were required to satisfy standard criteria: (1) reflect health status across multiple physiological systems; (2) increase in prevalence with age; (3) not be nearly universal in middle age; (4) have a baseline prevalence of at least 0.5%; and (5) have a missingness rate of no more than 5%. If two deficit variables were highly correlated (Spearman’s ρ >0.4), the one showing a stronger correlation with age was retained. This process ultimately resulted in 28 deficits, including medical conditions, symptoms, signs, and physical measurements. Each deficit was rescaled to a 0-1 range, with 0 indicating absence and 1 indicating presence. The frailty index was defined as the unweighted mean of the 28 variables. Based on the calculated frailty index, the population was divided into two groups using a cutoff value of 0.25 (corresponding to approximately 7 deficits): the robust group (Frailty index <0.25) and the frail group (Frailty index ≥0.25).

**3.2 Multimorbidity**

Multimorbidity was defined based on the number of distinct disease categories, using the International Classification of Diseases, 10th Revision (ICD-10) chapters as the classification framework. We excluded several ICD-10 chapters that are less relevant to chronic disease–related multimorbidity, including conditions of the sensory organs, perinatal and congenital disorders, as well as injuries and external causes. Specifically, the excluded chapters were (VII) diseases of the eye and adnexa; (VIII) diseases of the ear and mastoid process; (XII) diseases of the skin and subcutaneous tissue; (XIII) diseases of the musculoskeletal system and connective tissue; (XIV) diseases of the genitourinary system; (XV) pregnancy, childbirth and the puerperium; (XVI) certain conditions originating in the perinatal period; (XVII) congenital malformations, deformations and chromosomal abnormalities; (XVIII) symptoms, signs and abnormal clinical and laboratory findings, not elsewhere classified; (XIX) injury, poisoning and certain other consequences of external causes; (XX) external causes of morbidity and mortality; (XXI) factors influencing health status and contact with health services; and (XXII) codes for special purposes. Ultimately, diseases from 10 ICD-10 chapters were included in the analysis, as detailed in **Table B**. For each participant, all incident disease outcomes recorded from baseline to the third resurvey of CKB were ascertained and mapped to their respective ICD-10 chapters. The total number of unique chapters represented by these diseases was then counted to derive the disease count at the time of the third resurvey, which was used to quantify multimorbidity.

**Table B. Disease categories included in the multimorbidity analysis**

| **Chapter** | **Name** | **ICD-10 codes** |
| --- | --- | --- |
| I | Certain infectious and parasitic disease | A00-A99, B00-B99 |
| II | Neoplasms | C00-C97, D00-D48 |
| III | Diseases of the blood and blood-forming organs and certain disorders involving the immune mechanism | D50-D89 |
| IV | Endocrine, nutritional and metabolic diseases | E00-E90 |
| V | Mental and behavioural disorders | F00-F99 |
| VI | Diseases of the nervous system | G00-G99 |
| IX | Diseases of the circulatory system | I00-I99 |
| X | Diseases of the respiratory system | J00-J99 |
| XI | Diseases of the digestive system | K00-K93 |
| XIII | Diseases of the musculoskeletal system and connective tissue | M00-M99 |

**4. Validation and analysis**

**4.1 Cross-dataset validation**

We perform a cross-dataset validation using LAI by training it on CKB samples, model assessment for internal validation was performed using 10-fold cross-validation within the CKB training set, along with independent validation in the NHANES and VCTE cohorts. For assessment, the Pearson correlation coefficient (r) was calculated to assess the linear association between predicted and chronological age. The coefficient of determination (R²) was derived from linear regression models. Model accuracy was further evaluated using the root mean squared error (RMSE), computed as the square root of the mean of squared residuals, and the mean absolute error (MAE), calculated as the average of absolute residuals.

**4.2 Predictive analysis**

We validated whether LAI has better predictive power for aging-associated outcomes than does CA; Receiver operating characteristic (ROC) curves for mortality, liver-related events, and liver-related mortality. The predictive utility of the LAI is evaluated by calculating the area under the ROC curves (AUROC) and comparing its performance with that of the conventional assessment CA by Delong’s test.

**4.3 Survival analysis**

Kaplan-Meier survival curves were generated for both the training and validation datasets. In the CKB cohort, adjustments were made for sex, region, marital status, and education level; in the VCTE cohort, analyses were adjusted for sex and study center; in the NHANES cohort, adjustments were made for sex. Survival analyses were performed using log-rank tests.

**4.4 Association analysis**

Associations between LAI and clinical outcomes were examined using Cox proportional hazards models. In the CKB cohort, models were adjusted for sex, region, marital status, and education. In NHANES, weighted Cox proportional hazard models were used, adjusted for sex, ethnicity, marital status, and education. In the VCTE-Prognosis cohort, models were adjusted for sex and center. We also calculated the associations between LAA and clinical outcomes within subgroups defined by age, sex, and presence of diabetes.

**5. GWAS and Mendelian randomization**

**5.1 Genotyping and GWAS**

*(1) Genotyping in CKB*

Genotyping in the CKB was conducted using custom-designed Affymetrix Axiom arrays optimized for Chinese Han ancestry. The array was jointly developed by the University of Oxford’s Clinical Trial Service Unit and Epidemiological Studies Unit, the Beijing Genomics Institute, and Affymetrix (now Thermo Fisher Scientific) to provide genome-wide coverage suitable for the Chinese population. A 700K SNPs array was used to genotype approximately 32,000 participants, whereas a revised version with expanded coverage of ~800K SNPs was employed for around 69,000 participants. After quality control (call rate >0.98, plate effect *p*>10^-6^, batch effect *p*>10^-6^, HWE *p*>10^-6^ across 10 regions, and MAF differences <0.2 from 1000 Genomes East Asian frequencies), 532,415 biallelic variants common to both arrays were retained. The qualified genotypes for each chromosome were phased with SHAPEIT and imputation was performed for each 5-Mb interval with IMPUTE 4 based on haplotypes derived from the 1000 Genomes phase III. Additional quality control was applied to imputed SNPs, excluding those with (i) information measure (Info) ≤0.3 for MAF >3%, ≤0.6 for MAF 1–3%, ≤0.8 for MAF 0.5–1%, or ≤0.9 for MAF 0.1–0.5%; (ii) HWE p ≤10^-6^; (iii) call rate ≤95%; or (iv) location on sex chromosomes ^22^.

*(2) GWAS*

We identified LAA-associated variants in 15706 participants with available genotyping and LAA data. LAA was first rank-inverse normal transformed (RINT) based on the residuals obtained after regressing on age, age^2^, sex, and 10 study regions. GWAS was then performed using BOLT-LMM (version 2.3.4.) under an additive genetic model, which accounts for relatedness between individuals, adjusting for genotyping array version and 11 principal components (PCs). Independent SNPs were identified as those with *P*<1×10^-4^, r^2^<0.2, and distance >250 kb (based on EAS 1000 Genomes phase III) with PLINK 1.90. A weighted genetic risk score (GRS) for LAA was constructed by summing the number of LAA-increasing alleles.

**5.2 Mendelian randomization**

We used a two-stage least squares (2SLS) approach to assess the potential causal relationships between LAA and liver- related outcomes. First, we evaluated the validity of the GRS as the instrumental variable, with results shown in **Supplementary Table 4**. In the first stage, linear regression was performed with the GRS as the independent variable and LAA as the dependent variable to obtain genetically predicted LAA levels in the population, adjusting for sex, age, age^2^, 10 study regions, genotyping array version, and 11 PCs. In the second stage, Cox proportional hazards models or logistic regression models were used to evaluate the associations between genetically predicted LAA and each outcome, with the same covariate adjustments as in the first stage.

**Table C. MR assumptions and assessments** ^23^

| MR Assumptions | Assessments | Evidence of plausibility |
| --- | --- | --- |
| Relevance assumption: IVs are significantly associated with LAA | F-statistic and R^2^ | F-statistics = 2183.07;  R^2^ = 0.12 |
| Independence assumption: IVs have no associations with confounders | Examine associations between GRS and potential confounders | Figure B below |
| Exclusion restriction criteria (no pleiotropy): The risk of diseases is influenced only by LAA, not by other pathways. | MR egger intercept | Figure 5A |


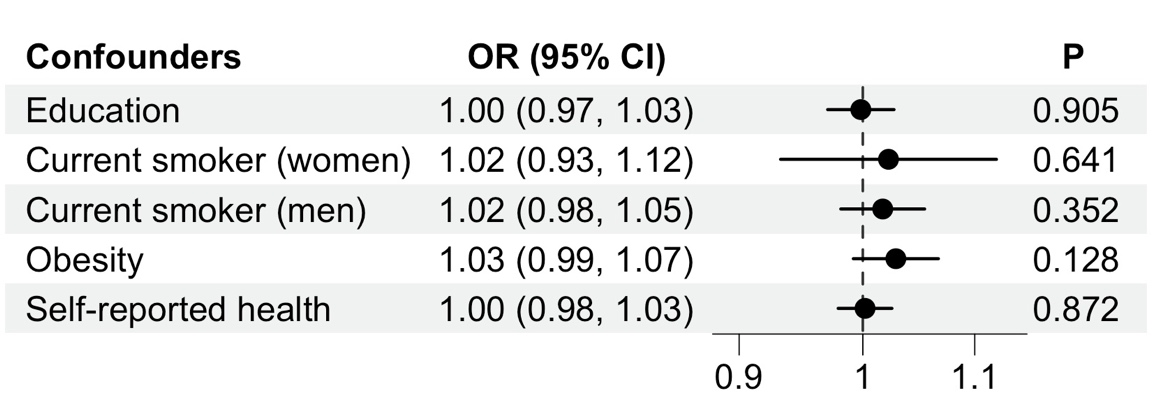


**Figure B. Associations between GRS of LAA and potential confounders**

Potential confounders were dichotomized as follows: education (middle school and higher vs. less than middle school), current smoker (yes vs. no), obesity (BMI ≥ 28 kg/m^2^ vs. BMI < 28 kg/m^2^), self-reported health (excellent/good vs. fair/poor).

**4.3** **Colocalization analysis**

We conducted colocalization analysis to determine whether LAA and liver-related outcomes shared the same genetic variants. LAA-related SNPs with *P*<1×10^-6^ were selected and cirrhosis and liver cancer were included in the analysis due to the availability of GWAS summary statistics in Biobank Japan^21^. Colocalization analysis employed a Bayesian model that estimates the posterior probabilities of five hypotheses (PPHs): (H0) no association with either LAA nor outcomes, (H1) association with LAA only, (H2) association with outcomes only, (H3) distinct causal variants underlying LAA and outcomes, and (H4) a shared causal variant underlying LAA and outcomes. The *coloc.abf* function in R package *coloc* was implemented with default prior probabilities (p1 = 1 × 10^-4^ for LAA, p2 = 1 × 10^-4^ for liver-related outcomes, and p12 = 1 × 10^-5^ for LAA and outcomes). Evidence for colocalization was defined as strong when PPH4 > 0.8^24^.

**4.4 Mendelian randomization with proteomics and pathway analysis**

In CKB, plasma level of 2923 unique proteins for 3977 participants were measured using the Olink Explore 3072 platform. We then explored genetical associations of LAA with these proteins by inverse-variance weighted MR using the *MendelianRandomization* R package. Genetic instruments for LAA were obtained from aforementioned GWAS with *P* ≤1×10^-4^. The effect estimates for associations of genetically predicted LAA with each protein were obtained with linear model, adjusted for sex, age, age^2^, 10 study regions, genotyping array version, 11 PCs and ascertainment. We additionally calculated the MR-Egger intercept to evaluate the presence of horizontal pleiotropy of instrumental variables. *P* values were corrected for multiple testing using the Benjamini-Hochberg approach. Proteins with FDR<0.05 were considered LAA-related and included for Gene Ontology (GO) enrichment analyses using the Database for Annotation, Visualization and Integrated Discovery (DAVID, <https://davidbioinformatics.nih.gov/>) ^25^.

**Reference**

1. Chen Z, Chen J, Collins R, et al. China Kadoorie Biobank of 0.5 million people: survey methods, baseline characteristics and long-term follow-up. *Int J Epidemiol*. 2011;40(6):1652-1666.

2. Guo Y, Yu C, Lv J, et al. Progress and achievements of large natural population cohort demonstration study. *Chin J Epidemiol*. 2023;(44):1-6.

3. Lin H, Lee HW, Yip TCF, et al. Vibration-Controlled Transient Elastography Scores to Predict Liver-Related Events in Steatotic Liver Disease. *JAMA*. 2024;331(15):1287-1297.

4. Man S, Deng Y, Ma Y, et al. Prevalence of liver steatosis and fibrosis in the general population and various high-risk populations: a nationwide study with 5.7 million adults in China. *Gastroenterology*. Published online June 26, 2023:S0016-5085(23)00928-9.

5. Xu Y, Liu Y, Cao Z, et al. Comparison of FibroTouch and FibroScan for staging fibrosis in chronic liver disease: Single-center prospective study. *Dig Liver Dis*. 2019;51(9):1323-1329.

6. Chen GF, Ping J, Gu HT, et al. [Correlation of liver stiffness measured by FibroTouch and FibroScan with Ishak fibrosis score in patients with chronic hepatitis B]. *Zhonghua Gan Zang Bing Za Zhi*. 2017;25(2):145-150.

7. National Center for Health Statistics. NHANES 2017-2018 Procedure Manuals. [2025-07-21]. Available from: https://wwwn.cdc.gov/nchs/nhanes/continuousnhanes/manuals.aspx?BeginYear=2017/2017_MEC_In-Person_Dietary_Interviewers_Manual.pdf.

8. Survey NHaNE. Survey NHaNE. Liver Ultrasound Transient Elastography Procedures Manual. Published online 2020.

9. K. Serum GGT Laboratory Procedure Manual. [2025-07-24]. Available from: https://wwwn.cdc.gov/nchs/data/nhanes/public/2017/labmethods/BIOPRO-J-MET-GGT-508.pdf.

10. Zhou XD, Lian L, Chen QF, et al. Effect of Hypertension on Long-term Adverse Clinical Outcomes and Liver Fibrosis Progression in MASLD. *J Hepatol*. Published online August 23, 2025:S0168-8278(25)02453-5.

11. Zhou XD, Kim SU, Yip TCF, et al. Long-term liver-related outcomes and liver stiffness progression of statin usage in steatotic liver disease. *Gut*. 2024;73(11):1883-1892.

12. Lin H, Lee HW, Yip TCF, et al. Vibration-Controlled Transient Elastography Scores to Predict Liver-Related Events in Steatotic Liver Disease. *JAMA*. 2024;331(15):1287-1297.

13. Mayer M. missRanger: Fast Imputation of Missing Values. Published online December 7, 2024.

14. Yang G, Rao C, Ma J, et al. Validation of verbal autopsy procedures for adult deaths in China. *Int J Epidemiol*. 2006;35(3):741-748.

15. Chen Z, Chen J, Collins R, et al. China Kadoorie Biobank of 0.5 million people: survey methods, baseline characteristics and long-term follow-up. *Int J Epidemiol*. 2011;40(6):1652-1666.

16. Rinella ME, Lazarus JV, Ratziu V, et al. A multisociety Delphi consensus statement on new fatty liver disease nomenclature. *J Hepatol*. 2023;79(6):1542-1556.

17. NCHS Data Linkage - Mortality Data - Public-Use Files. [2025-07-23]. Available from: https://www.cdc.gov/nchs/data-linkage/mortality-public.htm.

18. Levine ME, Lu AT, Quach A, et al. An epigenetic biomarker of aging for lifespan and healthspan. *Aging (Albany NY)*. 2018;10(4):573-591.

19. Fong S, Pabis K, Latumalea D, et al. Principal component-based clinical aging clocks identify signatures of healthy aging and targets for clinical intervention. *Nat Aging*. 2024;4(8):1137-1152.

20. Aging Biomarker Consortium, Jiang M, Zheng Z, et al. A biomarker framework for liver aging: the Aging Biomarker Consortium consensus statement. *Life Medicine*. 2024;3(1):lnae004.

21. Fan J, Yu C, Guo Y, et al. Frailty index and all-cause and cause-specific mortality in Chinese adults: a prospective cohort study. *The Lancet Public health* 2020;5(12):e650-e60.

22. Sakaue S, Kanai M, Tanigawa Y, et al. A cross-population atlas of genetic associations for 220 human phenotypes. *Nat Genet*. 2021;53(10):1415-1424.

23. Emdin CA, Khera AV, Kathiresan S. Mendelian Randomization. *JAMA*. 2017;318(19):1925-1926.

24. Wallace C. A more accurate method for colocalisation analysis allowing for multiple causal variants. *PLoS Genet*. 2021;17(9):e1009440.

25. Dunn J, Ferluga S, Sharma V, et al. Proteomic analysis discovers the differential expression of novel proteins and phosphoproteins in meningioma including NEK9, HK2 and SET and deregulation of RNA metabolism. *EBioMedicine*. 2019;40:77-91.

# Supplementary Table 1. Transparent Reporting of a multivariable prediction model for Individual Prediction or Diagnosis (TRIPOD) guidelines

| **Section/Topic** | **Item** | **Development** | **Checklist item** | **Page** |
| --- | --- | --- | --- | --- |
| **TITLE** | | | | |
| *Title* | 1 | D;E | Identify the study as developing or evaluating the performance of a multivariable prediction model, the target population, and the outcome to be predicted | 1 |
| **ABSTRACT** | | | | |
| *Abstract* | 2 | D;E | See TRIPOD+AI for Abstracts checklist | 3 |
| **INTRODUCTION** | | | | |
| *Background* | 3a | D;E | Explain the healthcare context (including whether diagnostic or prognostic) and rationale for developing or evaluating the prediction model, including references to existing models | 4 |
|  | 3b | D;E | Describe the target population and the intended purpose of the prediction model in the context of the care pathway, including its intended users (e.g., healthcare professionals, patients, public) | 4-5 |
|  | 3c | D;E | Describe any known health inequalities between sociodemographic groups | 4-5 |
| *Objectives* | 4 | D;E | Specify the study objectives, including whether the study describes the development or validation of a prediction model (or both) | 5 |
| **METHODS** | | | | |
| *Data* | 5a | D;E | Describe the sources of data separately for the development and evaluation datasets (e.g., randomised trial, cohort, routine care or registry data), the rationale for using these data, and representativeness of the data | 5-7 |
|  | 5b | D;E | Specify the dates of the collected participant data, including start and end of participant accrual; and, if applicable, end of follow-up | 6-8 |
| *Participants* | 6a | D;E | Specify key elements of the study setting (e.g., primary care, secondary care, general population) including the number and location of centres | 5-8 |
|  | 6b | D;E | Describe the eligibility criteria for study participants | 5 |
|  | 6c | D;E | Give details of any treatments received, and how they were handled during model development or evaluation, if relevant | Not applicable |
| *Data preparation* | 7 | D;E | Describe any data pre-processing and quality checking, including whether this was similar across relevant sociodemographic groups | 8-10 |
| *Outcome* | 8a | D;E | Clearly define the outcome that is being predicted and the time horizon, including how and when assessed, the rationale for choosing this outcome, and whether the method of outcome assessment is consistent across sociodemographic groups | 7-8 |
|  | 8b | D;E | If outcome assessment requires subjective interpretation, describe the qualifications and demographic characteristics of the outcome assessors | 7-8 |
|  | 8c | D;E | Report any actions to blind assessment of the outcome to be predicted | Not applicable |
| *Predictors* | 9a | D | Describe the choice of initial predictors (e.g., literature, previous models, all available predictors) and any pre-selection of predictors before model building | 8-9 |
|  | 9b | D;E | Clearly define all predictors, including how and when they were measured (and any actions to blind assessment of predictors for the outcome and other predictors) | 9 |
|  | 9c | D;E | If predictor measurement requires subjective interpretation, describe the qualifications and demographic characteristics of the predictor assessors | 9 |
| *Sample size* | 10 | D;E | Explain how the study size was arrived at (separately for development and evaluation), and justify that the study size was sufficient to answer the research question. Include details of any sample size calculation | 5-7 |
| *Missing data* | 11 | D;E | Describe how missing data were handled. Provide reasons for omitting any data | 6 |
| *Analytical methods* | 12a | D | Describe how the data were used (e.g., for development and evaluation of model performance) in the analysis, including whether the data were partitioned, considering any sample size requirements | 8-10 |
|  | 12b | D | Depending on the type of model, describe how predictors were handled in the analyses (functional form, rescaling, transformation, or any standardisation). | 8-10 |
|  | 12c | D | Specify the type of model, rationale^2^, all model-building steps, including any hyperparameter tuning, and method for internal validation | 8-11 |
|  | 12d | D;E | Describe if and how any heterogeneity in estimates of model parameter values and model performance was handled and quantified across clusters (e.g., hospitals, countries). See TRIPOD-Cluster for additional considerations^3^ | 9-10 |
|  | 12e | D;E | Specify all measures and plots used (and their rationale) to evaluate model performance (e.g., discrimination, calibration, clinical utility) and, if relevant, to compare multiple models | 9-10 |
|  | 12f | E | Describe any model updating (e.g., recalibration) arising from the model evaluation, either overall or for particular sociodemographic groups or settings | 9-10 |
|  | 12g | E | For model evaluation, describe how the model predictions were calculated (e.g., formula, code, object, application programming interface) | 9-10 |
| *Class imbalance* | 13 | D;E | If class imbalance methods were used, state why and how this was done, and any subsequent methods to recalibrate the model or the model predictions | Not applicable |
| *Fairness* | 14 | D;E | Describe any approaches that were used to address model fairness and their rationale | 9-10 |
| *Model output* | 15 | D | Specify the output of the prediction model (e.g., probabilities, classification). Provide details and rationale for any classification and how the thresholds were identified | 9-10 |

# Supplementary Table 2. ICD-10 codes used to define liver-related events and mortality

| **ICD-10 code** |
| --- |
| **LRE** |
| C22.0, C22.7; K70.1, K70.2, K70.3, K70.4, K70.9; K71.7, K71.9; K72.0, K72.1, K72.9; K73.0, K73.2, K73.8, K73.9; K74.0, K74.1, K74.2; K75.8, K75.9; K76.6, K76.7, K76.8, K76.9; I85.0, I85.9, I98.2, I98.3 |
| **LRM** |
| The same ICD-10 codes as LRE where these were the underlying cause of death on the death certificates. |

Abbreviations: LRE, liver-related events; LRM: liver-related mortality.

# Supplementary Table 3. Metrics of LAI in the development and validation cohorts

|  | **Mean (SD),**  **years** | **RMSE, years** | **LAI Accel SD, years** | **Pearson *r*** |
| --- | --- | --- | --- | --- |
| Training cohort (CKB) |  |  |  |  |
| CA | 65.27 (9.02) |  |  |  |
| LAI-13 | 65.27 (9.31) | 1.88 | 1.95 | 0.978 |
| LAI-5 | 65.32 (9.28) | 2.21 | 2.27 | 0.970 |
| Validation cohort (NHANES) |  |  |  |  |
| CA | 63.23(10.62) |  |  |  |
| LAI-13 | 63.25 (10.95) | 2.19 | 2.26 | 0.979 |
| LAI-5 | 63.31 (10.66) | 2.61 | 2.64 | 0.969 |
| Validation cohort (VCTE-Prognosis cohort) | |  |  |  |
| CA | 59.13(8.61) |  |  |  |
| LAI-13 | 52.81 (13.86) | 2.11 | 2.36 | 0.970 |
| LAI-5 | 52.93 (13.55) | 2.72 | 2.91 | 0.949 |

# Supplementary Table 4. Calibration results of LAI for liver-related events

| **Events** | **Index** | **Brier score** | **Slope** | **ICI** | **E50** | **E90** |
| --- | --- | --- | --- | --- | --- | --- |
| **CKB** |  |  |  |  |  |  |
| LRE | CA | 0.0136 (0.0121, 0.0152) | 1.0000 | 0.0003 | 0.0003 | 0.0005 |
| LRE | LAI-13 | 0.0136 (0.0121, 0.0151) | 1.0000 | 0.0004 | 0.0004 | 0.0006 |
| LRE | LAI-5 | 0.0136 (0.0121, 0.0151) | 1.0000 | 0.0004 | 0.0004 | 0.0006 |
| LRM | CA | 0.0006 (0.0003, 0.0009) | 1.0000 | 0.0004 | 0.0003 | 0.0005 |
| LRM | LAI-13 | 0.0006 (0.0003, 0.0009) | 1.0000 | 0.0002 | 0.0002 | 0.0003 |
| LRM | LAI-5 | 0.0006 (0.0003, 0.0009) | 1.0000 | 0.0002 | 0.0001 | 0.0002 |
| **VCTE-Prognosis cohort** | | | | | | |
| LRE | CA | 0.0279 (0.0247, 0.0311) | 1.0000 | 0.0023 | 0.0022 | 0.0034 |
| LRE | LAI-13 | 0.0274 (0.0243, 0.0305) | 1.0000 | 0.0025 | 0.0016 | 0.0061 |
| LRE | LAI-5 | 0.0271 (0.0240, 0.0302) | 1.0000 | 0.0019 | 0.0012 | 0.0050 |

Abbreviations: CA, chronological age; ICI, integrated calibration index; LAI, liver aging index; LRE, liver-related events; LRM: liver-related mortality.

# Supplementary Table 5. Colocalization analysis of LAI in Biobank Japan

| **Outcome** | **rsid** | **No. SNP** | **PP.H0.abf** | **PP.H1.abf** | **PP.H2.abf** | **PP.H3.abf** | **PP.H4.abf** |
| --- | --- | --- | --- | --- | --- | --- | --- |
| Cirrhosis | rs5751904 | 1516 | 6.31E-09 | 4.77E-10 | 9.07E-01 | 6.85E-02 | 2.44E-02 |
|  | rs11066001 | 640 | 3.56E-17 | 5.07E-07 | 3.73E-13 | 4.32E-03 | 9.96E-01 |
|  | rs12231737 | 721 | 1.12E-16 | 7.72E-07 | 1.31E-12 | 8.04E-03 | 9.92E-01 |
|  | rs78675159 | 1725 | 1.20E-03 | 9.76E-05 | 8.77E-01 | 7.11E-02 | 5.03E-02 |
|  | rs3020216 | 3208 | 4.80E-04 | 6.84E-05 | 8.62E-01 | 1.23E-01 | 1.43E-02 |
|  | rs7412 | 1210 | 1.43E-02 | 9.64E-04 | 8.89E-01 | 6.00E-02 | 3.53E-02 |
|  | rs7554936 | 782 | 2.17E-01 | 1.01E-02 | 7.18E-01 | 3.34E-02 | 2.19E-02 |
|  | rs57904317 | 1570 | 1.90E-01 | 1.28E-02 | 6.86E-01 | 4.60E-02 | 6.44E-02 |
|  | rs143068247 | 1795 | 2.20E-01 | 2.44E-02 | 6.44E-01 | 7.16E-02 | 4.01E-02 |
| Liver cancer | rs5751904 | 1516 | 6.13E-09 | 6.34E-10 | 8.81E-01 | 9.11E-02 | 2.77E-02 |
|  | rs11066001 | 640 | 3.74E-08 | 5.16E-07 | 3.92E-04 | 4.42E-03 | 9.95E-01 |
|  | rs12231737 | 721 | 4.09E-08 | 1.02E-06 | 4.78E-04 | 1.09E-02 | 9.89E-01 |
|  | rs78675159 | 1725 | 1.10E-03 | 1.58E-04 | 8.01E-01 | 1.15E-01 | 8.27E-02 |
|  | rs3020216 | 3208 | 4.65E-04 | 8.24E-05 | 8.36E-01 | 1.48E-01 | 1.52E-02 |
|  | rs7412 | 1210 | 1.39E-02 | 1.07E-03 | 8.67E-01 | 6.63E-02 | 5.16E-02 |
|  | rs7554936 | 782 | 2.14E-01 | 9.11E-03 | 7.06E-01 | 3.01E-02 | 4.10E-02 |
|  | rs57904317 | 1570 | 1.89E-01 | 1.77E-02 | 6.81E-01 | 6.36E-02 | 4.88E-02 |
|  | rs143068247 | 1795 | 2.18E-01 | 2.67E-02 | 6.40E-01 | 7.82E-02 | 3.70E-02 |

The analysis assessed five competing hypotheses: (H0) no association with either trait; (H1) association with trait 1 only; (H2) association with trait 2 only; (H3) distinct causal variants associated with two traits; and (H4) a shared causal variant associated with both traits.

Abbreviation: PP, posterior probability; SNP, single nucleotide polymorphism.

# Supplementary Table 6. Genetic associations of LAA with other diseases and biomarkers

| **Diseases** | **HR (95% CI)** | ***p*-value** | **MR Egger intercept *p*-value** |  | **Biomarkers** | **β (95% CI)** | ***p*-value** | **MR Egger intercept *p*-value** |
| --- | --- | --- | --- | --- | --- | --- | --- | --- |
| **Disease** |  |  |  |  | **Biochemistry** |  |  |  |
| Intracerebral haemorrhage | 1.08 (1.02, 1.14) | 0.004 | <0.001 |  | UA | 9.25 (4.49, 14.01) | <0.001 | 0.204 |
| Cerebral infarction | 1.04 (1.01, 1.08) | 0.023 | 0.198 |  | **Protein*** |  |  |  |
| Atrial fibrillation | 1.22 (1.03, 1.45) | 0.020 | 0.058 |  | Olink_APP | -0.13 (-0.22, -0.04) | 0.005 | 0.446 |
| Primary hypertension | 1.07 (1.03, 1.10) | <0.001 | 0.014 |  | SomaScan_APP | -0.15 (-0.23, -0.06) | <0.001 | 0.311 |
| Gout | 1.40 (1.19, 1.66) | <0.001 | 0.088 |  |  |  |  |  |

Abbreviation: APP, amyloid precursor proteins; UA, uric acid.

*Olink_APP denotes amyloid precursor protein measured by the Olink platform. SomaScan_APP denotes amyloid precursor protein measured by the Somalogic platform.

# Supplementary Table 7. Genetic associations of LAA with plasma proteins

| **Protein** | **β (95% CI)** | **FDR**  ***p*-value** | **MR Egger Intercept *p*-value** |
| --- | --- | --- | --- |
| TMPRSS11D | 0.22 (0.13, 0.31) | 0.002 | 0.658 |
| VWA1 | 0.25 (0.14, 0.36) | 0.006 | 0.065 |
| PLXNB2 | 0.20 (0.11, 0.29) | 0.006 | 0.192 |
| VCAN | 0.23 (0.13, 0.33) | 0.006 | 0.546 |
| CCL13 | -0.18 (-0.27, -0.10) | 0.006 | 0.598 |
| ALDH2 | 0.26 (0.14, 0.38) | 0.010 | 0.071 |
| PON2 | 0.21 (0.11, 0.31) | 0.013 | 0.097 |
| TEK | 0.16 (0.08, 0.23) | 0.013 | 0.467 |
| SLAMF7 | 0.19 (0.10, 0.28) | 0.015 | 0.186 |
| SIGLEC1 | 0.16 (0.08, 0.25) | 0.021 | 0.872 |
| CCN2 | -0.18 (-0.27, -0.09) | 0.023 | 0.664 |
| MSR1 | 0.17 (0.08, 0.25) | 0.023 | 0.122 |
| TXNL1 | 0.19 (0.10, 0.29) | 0.027 | 0.190 |
| ARHGEF5 | 0.16 (0.08, 0.25) | 0.046 | 0.532 |
| ANGPT1 | -0.16 (-0.25, -0.07) | 0.048 | 0.865 |
| C1QTNF1 | 0.15 (0.07, 0.24) | 0.048 | 0.481 |
| EPCAM | 0.17 (0.08, 0.27) | 0.048 | 0.923 |
| MARCO | 0.16 (0.08, 0.25) | 0.048 | 0.217 |
| AFAP1 | -0.17 (-0.27, -0.08) | 0.048 | 0.284 |
| GRPEL1 | 0.17 (0.08, 0.26) | 0.048 | 0.707 |
| HBEGF | -0.16 (-0.25, -0.07) | 0.049 | 0.503 |
| CTSD | 0.17 (0.08, 0.27) | 0.049 | 0.684 |

# Supplementary Table 8. Information of previous studies on Liver Biological Age

| **Reference** | **Indicators, prediction phenotype** | **Methods** | **Training N (age), dataset** | **Validation N, dataset** | **Correlation with CA,r** | **Associations with LRE** | **Associations with Mortality** | |
| --- | --- | --- | --- | --- | --- | --- | --- | --- |
|  |  |  |  |  |  |  | **HR (95%CI)** | **AUROC** |
| **Liver biological age** |  |  |  |  |  |  |  |  |
| Liver image age^1^ | Liver MRI imaging data; CA | Convolutional neural network | 43,267 (37-82) | - | 0.85 | r: -0.015-0.008 | - | - |
| Liver biological age^2^ | 4 blood biochemical markers; CA | KDM | 3,193 (≥18) | - | - | - | - | - |
| Hepatic age^3^ | 9 blood biochemical markers; CA | Linear support vector machine | 143,423 (39-73) | internal-validation | 0.32/0.45* (M/F) | - | 1.08(1.05-1.10)* | 0.750 |
| **Liver biological age in multi-organ system-specific aging studies** | | | |  |  |  |  |  |
| Liver dysregulation ageotype^4^ | Blood biochemical tests and multi-omics; CA | Linear regression model and clustering analysis | 106 (29-75) | - | - | - | - | - |
| Liver age scores^5^ | 6 blood biochemical markers and DNA methylation; Mortality | Principal component analysis and elastic-net Cox | 5,127 (51-100) | 5,600, three cohorts of WHI | - | - | 1.13 (1.08, 1.17)* | - |
| Liver biological age^6^ | 47 blood biochemical and metabolomic markers; CA | KDM | 2,599 (20-45) | 3,868, NHANES | 0.37 | Associated with non-alcoholic fatty liver | 1.60 (1.50-1.70)^†^ | - |
| Liver age^7^ | 113 blood proteins; CA | LASSO regression | 1,398 (27-101) | 888, LonGenity | 0.72 | - | 1.29 (1.16, 1.44)^†^ | - |
| Liver aging models^8^ | 79 blood proteins; CA | Elastic-net Cox proportional hazards | 44,952 (40-70) | 1,031-1,463,3 proteomic datasets | 0.62 | HR:  1.30 (1.15, 1.47) | 1.22 (1.19, 1.26) | - |
|  | 79 blood proteins; mortality |  |  |  | 0.40* | 2.30 (2.11, 2.51)^†^ | 1.72 (1.68, 1.76)^†^ |  |

Abbreviation: BA, biological age; CA, chronological age; HRS, the Health and Retirement Study; WHI, the Women’s Health Initiative.

^†^ are expressed as hazard ratios (HRs) per standard deviation (SD) increase. * An asterisk indicates R^2^.

1. Le Goallec A, Diai S, Collin S, Prost JB, Vincent T, Patel CJ. Using deep learning to predict abdominal age from liver and pancreas magnetic resonance images. *Nat Commun*. 2022;13(1):1979.

2. Xing W, Gao W, Zhao Z, et al. Dietary flavonoids intake contributes to delay biological aging process: analysis from NHANES dataset. *J Transl Med*. 2023;21(1):492.

3. Tian YE, Cropley V, Maier AB, Lautenschlager NT, Breakspear M, Zalesky A. Heterogeneous aging across multiple organ systems and prediction of chronic disease and mortality. *Nat Med*. 2023;29(5):1221-1231.

4. Ahadi S, Zhou W, Schüssler-Fiorenza Rose SM, et al. Personal aging markers and ageotypes revealed by deep longitudinal profiling. *Nat Med*. 2020;26(1):83-90.

5. Sehgal R, Markov Y, Qin C, et al. Systems Age: a single blood methylation test to quantify aging heterogeneity across 11 physiological systems. *Nat Aging*. 2025;5(9):1880-1896.

6. Nie C, Li Y, Li R, et al. Distinct biological ages of organs and systems identified from a multi-omics study. *Cell Rep*. 2022;38(10):110459.

7. Oh HSH, Rutledge J, Nachun D, et al. Organ aging signatures in the plasma proteome track health and disease. *Nature*. 2023;624(7990):164-172.

8. Goeminne LJE, Vladimirova A, Eames A, et al. Plasma protein-based organ-specific aging and mortality models unveil diseases as accelerated aging of organismal systems. *Cell Metab*. 2025;37(1):205-222.e6.

# Supplementary Figure 1. Correlation structure among the features for the LAI in the construction and validation cohorts


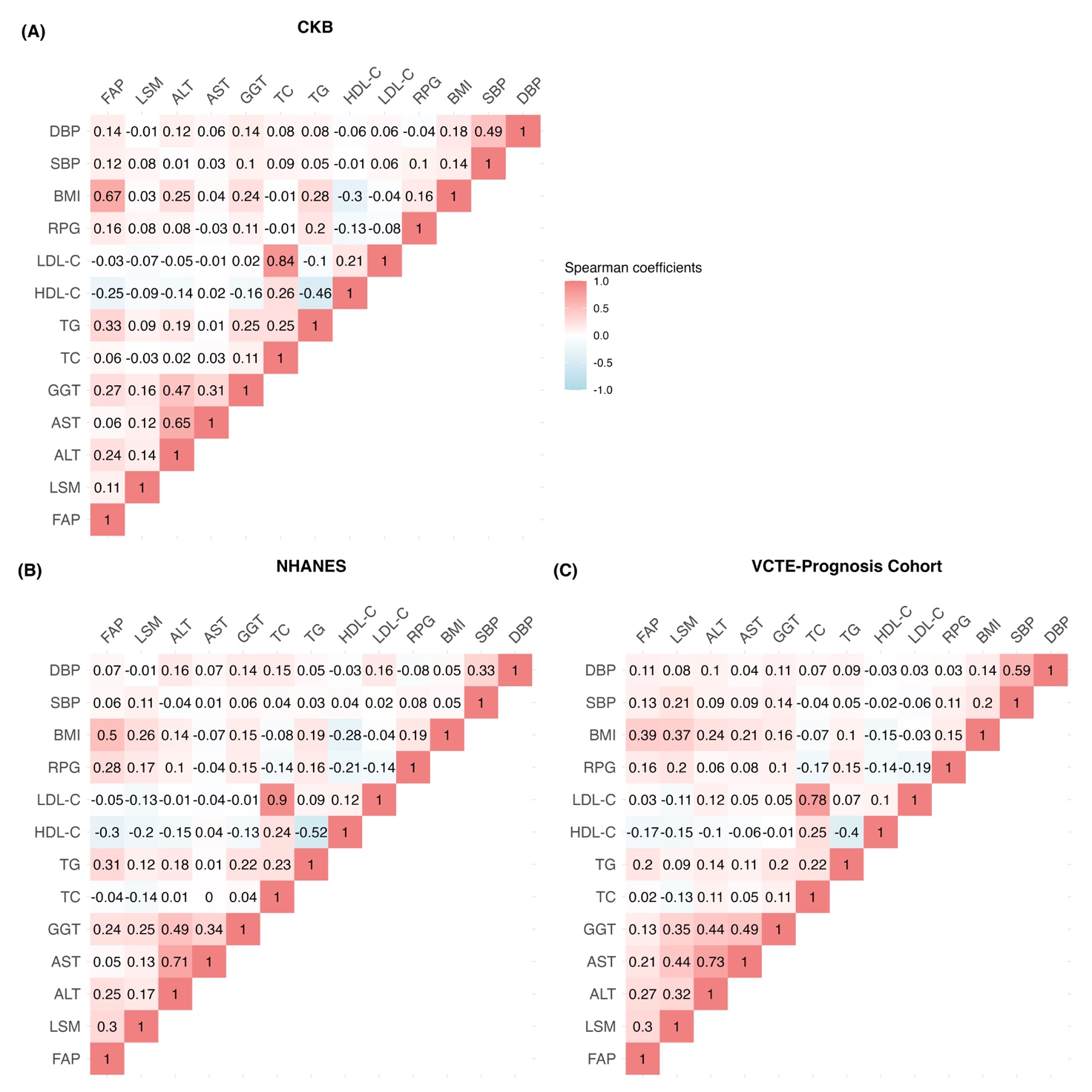


This figure shows Spearman correlation coefficients among biomarkers in three cohorts (CKB, NHANES, and VCTE-Prognosis cohort). Analyzed biomarkers include 13 biomarkers for LAI construction.

Abbreviation: RPG, random plasma glucose; TC, total cholesterol; TG, triglycerides; HDL-C; high-density lipoprotein cholesterol; LDL-C, low-density lipoprotein cholesterol; FAP, fat attenuation parameter; LSM, liver stiffness measurement; BMI，body mass index；SBP, systolic blood pressure; DBP, diastolic blood pressure.

# Supplementary Figure 2. Ridgeline plots of development and validation populations binned by decade for CA


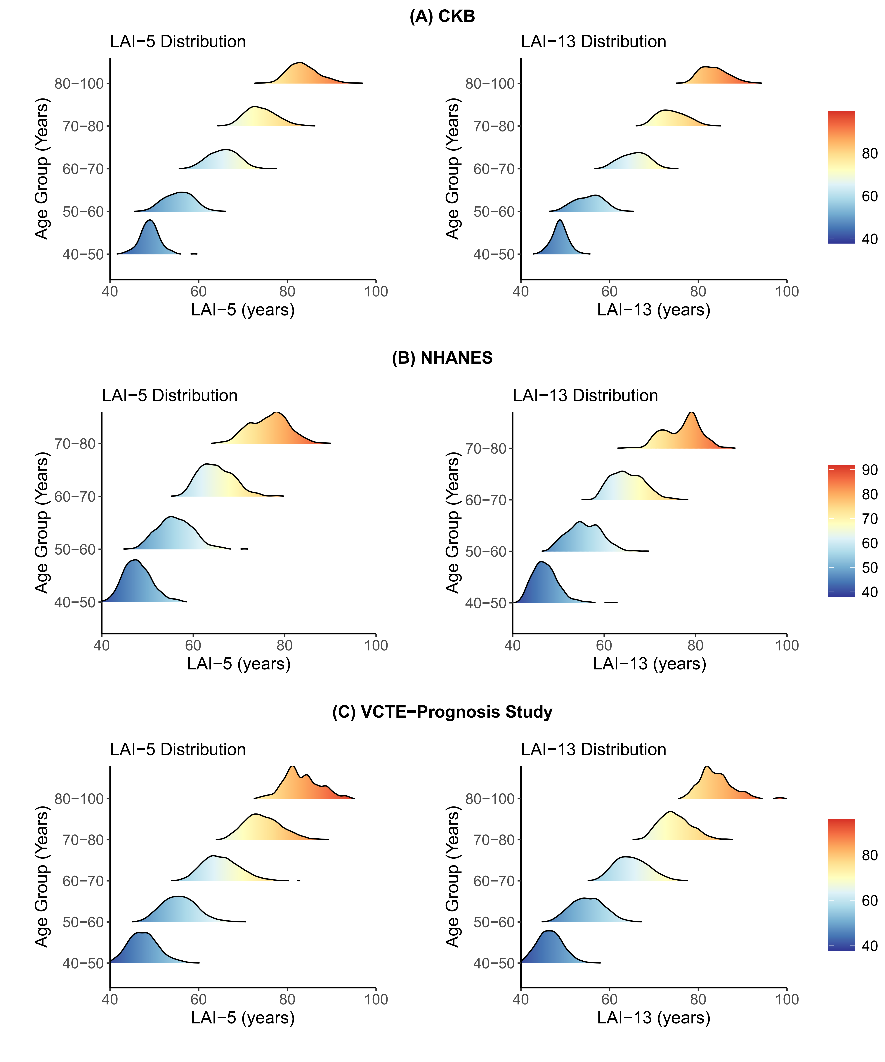


The distribution of LAI-5 and LAI-13 ridgeline plots of age groups grouped by decade for CA. Color gradients (from blue to red) correspond to the density distribution of LAI values within each age group. Each cohort panel includes LAI-5 distribution and LAI-13 distribution.

# Supplementary Figure 3. Model performance of LAI for liver-related outcome


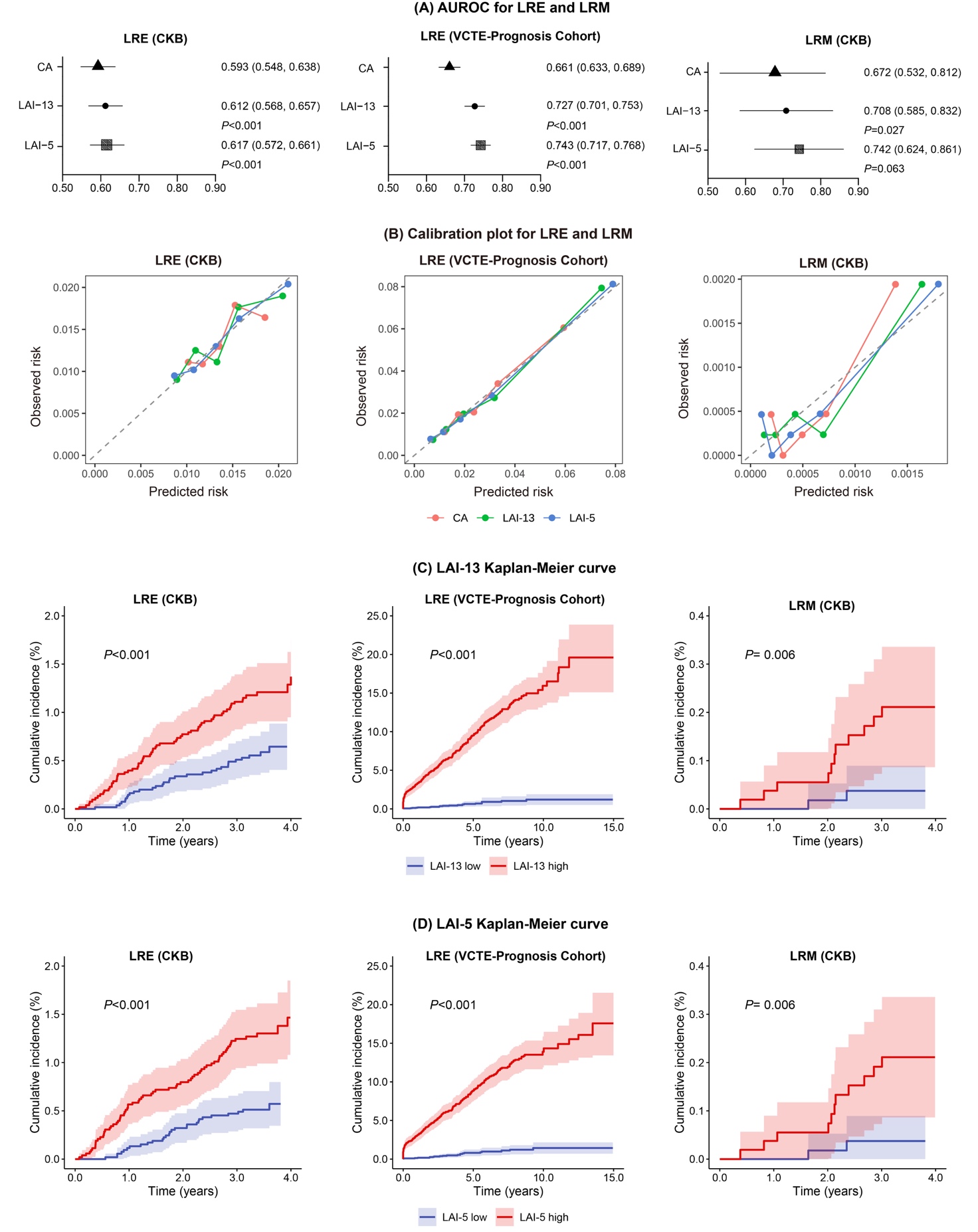


Abbreviation: CA, chronological age; LAI, liver aging index.

Panel A shows comparison of AUROCs between LAI and CA using DeLong’s test.

Panel B shows the calibration curves for CA, LAI-13, and LAI-5.

Panel C and panel D show Kaplan-Meier survival curve where subjects are stratified by selecting the highest (worst) and lowest (best) 25% of LAA. The analyses were adjusted for age, sex, regions (CKB and VCTE-Prognosis cohort), education, and marital status. All P values were calculated using the log-rank test for comparing survival distributions between groups.

# Supplementary Figure 4. Associations of LAI with all-cause mortality and liver-related outcomes in CKB participants without and with MASLD


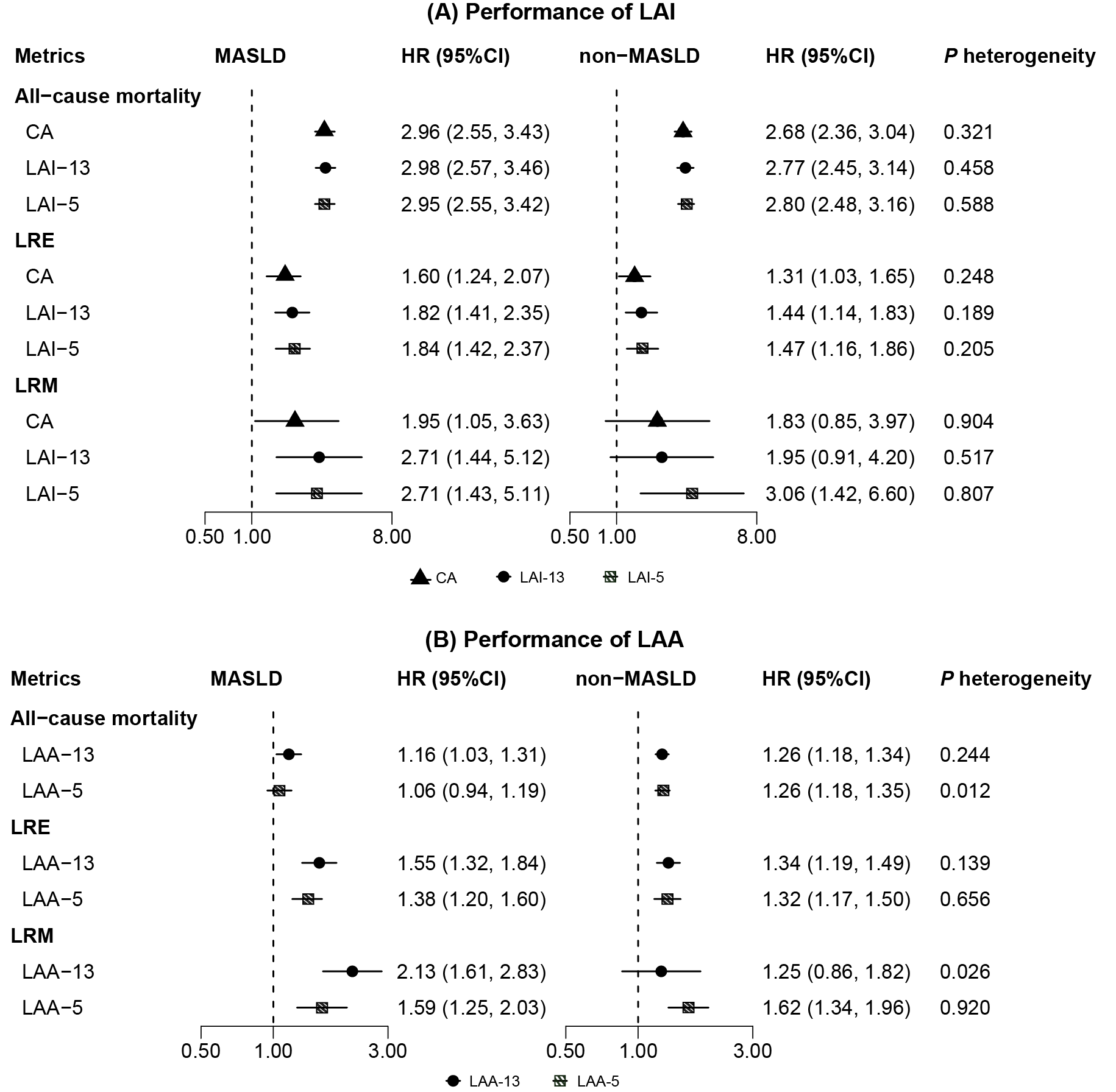


The results were obtained through Cox regression model calculation in CKB, adjusting for age, sex, regions, education, and marital status.

# Supplementary Figure 5. Associations of LAI and LAA with geriatric indicators in CKB


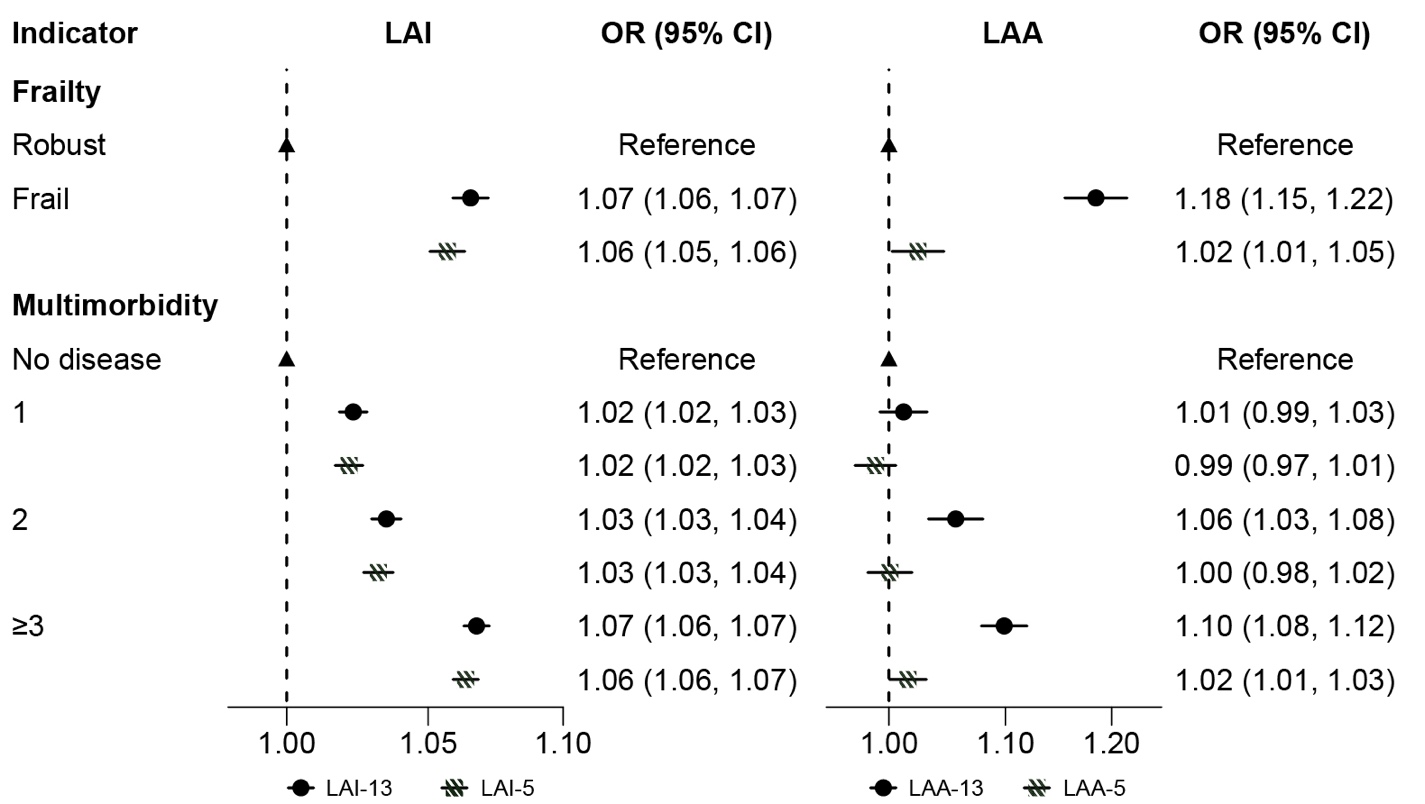


The results were estimated using binary or multinomial logistic regression models in the CKB, adjusting for age, sex, region, education, and marital status, as appropriate.
